# Synergistic Enhancement of Ethanol Oxidation on Pd/Ce_2_Ti_2_O_7_–TiO_2_ via Multiorbital (p–d–f) Interactions

**DOI:** 10.1021/acs.inorgchem.5c04103

**Published:** 2025-12-09

**Authors:** Refiloe Modise, Patrick V. Mwonga, Jeseelan Pillay, Kenneth I. Ozoemena

**Affiliations:** † Molecular Sciences Institute, School of Chemistry, 37707 University of the Witwatersrand, Private Bag 3, PO Wits, Johannesburg 2050, South Africa; ‡ National Metrology Institute of South Africa, Private Bag X34, Lynnwood Ridge 0040, South Africa

## Abstract

Ethanol oxidation reaction (EOR) is a key component of direct ethanol fuel cells (DEFCs). However, due to its slow kinetics, EOR remains a significant challenge, leading to an increasing search for low-cost, efficient, and stable catalysts. Herein, palladium (Pd) nanostructured catalysts supported on titanium dioxide (Pd/TiO_2_) and cerium titanate (Pd/Ce_2_Ti_2_O_7_–TiO_2_) were prepared for EOR. The Pd/Ce_2_Ti_2_O_7_–TiO_2_ catalyst exhibits superior electroactivity toward the EOR, demonstrating a high mass activity of 2.70 and 2.63-fold relative to Pd/TiO_2_ and Pd/C, respectively. The enhanced behavior was attributed to the bifunctional mechanism, improved electron transport, and downshifted d-band center (ε_d_). Density functional theory (DFT) calculations show a higher density of states for Pd/Ce_2_Ti_2_O_7_–TiO_2_,suggesting higher EOR kinetics than the Pd and Pd/TiO_2_, due to its multiorbital (p–d–f) hybridization. The accelerated durability test (ADT) reveals that the Pd/Ce_2_Ti_2_O_7_–TiO_2_ catalyst exhibits satisfactory durability, showing an electrochemically active surface area (ECSA) loss of 18.8% compared with the Pd/C catalyst (33.3% loss) after 1000 cycles. Furthermore, the Pd/Ce_2_Ti_2_O_7_–TiO_2_ catalyst exhibited a low detection limit of 12.0 μM toward the nonenzymatic amperometric detection of ethanol. Thus, this study demonstrates the promising use of Ce_2_Ti_2_O_7_–TiO_2_ as a support for Pd-based catalysts for EOR in DEFCs and a nonenzymatic amperometric sensor.

## Graphical abstract

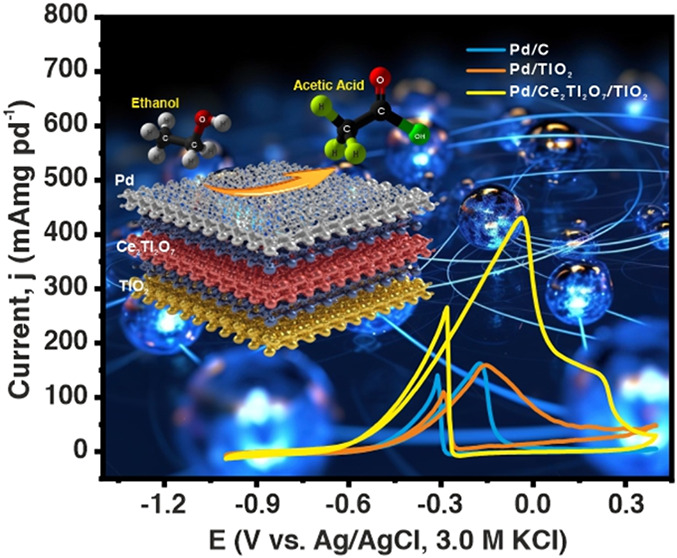

## 1 Introduction

Direct ethanol fuel cells (DEFCs) convert the chemical energy of ethanol and oxygen into electrical energy through electrochemical processes in the anodic and cathodic compartments, respectively.^1^ These devices have gained increased research interest due to their attractive properties, such as reduced carbon dioxide (CO_2_) emissions and the use of a sustainable fuel source, ethanol, which is environmentally benign and can be produced on a large scale from renewable resources (biomass).^2–4^ DEFCs are applicable in stationary, transportation, and portable devices. Unlike hydrogen (H_2_), low-molecular-weight alcohols, such as methanol and ethanol, are the most preferred fuel sources due to their ease of storage and transportation. In particular, the use of ethanol is desirable compared to methanol due to its higher energy density (8.01 kWh kg^–1^) relative to methanol’s (6.09 kWh kg^–1^) and lower toxicity.^5–7^ Ideally, it is required that ethanol be completely oxidized via the 12 electrons to produce carbonate (CO_3_^2–^) in an alkaline electrolyte to achieve the maximum output.

However, there are challenges associated with the complete oxidation of ethanol, such as the sluggish kinetics, catalyst poisoning, and poor stability.^3,8,9^ Platinum (Pt) catalysts are widely employed in acidic and alkaline electrolytes for ethanol oxidation reactions (EORs). However, due to the high cost and depleting reserves, the large-scale commercialization of DEFCs is limited.^10^ Also, Pt nanoparticles are highly susceptible to catalyst poisoning by the reaction intermediate species, such as the acetyl group (CH_3_CO_ads_) or carbon monoxide (CO_ads_).^11–13^ In this regard, developing and investigating low-cost, robust, and highly efficient catalysts for DEFCs remains a key research topic.^14^ Although palladium (Pd) belongs to the platinum group metals (PGMs), it is more abundant and lower in cost compared to Pt.^15^ Furthermore, the use of Pd has shown enhanced catalytic properties for EOR in alkaline electrolyte and is less prone to catalyst poisoning.^16,17^ Like Pt, the quantity of Pd can be reduced by the stoichiometric addition of nonprecious metals like tin (Sn),^18,19^ nickel (Ni),^20–22^ copper (Cu),^23,24^ among others, serving as cocatalysts to promote the electrocatalytic reaction while reducing the cost of the catalyst.^6,25–30^ Moreover, state-of-the-art catalysts in DEFCs use low-cost and abundant carbon as the support material. However, the use of carbon is often associated with electrochemical degradation at the DEFCs’ operating temperatures, leading to agglomeration of the supported catalyst and reduced electrocatalyst efficiency and stability.^31^ This accounts for the increasing research interest toward corrosion-resistant materials such as titanium dioxide (TiO_2_),^17,32,33^ tungsten oxide (WO_3_),^34,35^ cerium oxide (CeO_2_),^36–38^ among others, as supporting materials for metal nanoparticles. Transition metal oxides are also distinguished for their strong interaction between the metal (catalyst) and support, as well as their high oxophilicity.^17,34,39^ The strong metal–support interaction (SMSI) refers to the optimal interaction between a metal catalyst and its support. This phenomenon accounts for enhanced electrocatalytic properties by inducing an electronic effect on the catalyst, subsequently resulting in a downshifted d-band center (ε_d_), which favors the weak adsorption of the reaction intermediate species on the catalyst’s surface, making it more tolerant to poisoning, thus improving its stability.^40,41^ In addition, the enhanced interaction stabilizes the supported nanoparticles by preventing their agglomeration during the reaction, increasing the longevity of the catalysts.^42^ On the other hand, due to their high oxophilic nature, metal oxides facilitate the formation of hydroxyl species (OH) at low overpotentials, which subsequently adsorb on metallic surfaces (M–OH_ads_). The M–OH_ads_ species facilitates the removal of reaction intermediates on the adjacent Pd nanoparticles, a phenomenon known as the bifunctional mechanism.^11,43^ Notwithstanding that, the low surface area and poor electronic conductivity of metal oxides compared to carbon materials limit the commercialization of metal-oxide supported catalysts as electrocatalysts for EOR.^44–48^ To address these issues, several studies have demonstrated the preparation of substoichiometric metal oxides (M_*x*_O_2*x*–1_). Particularly with TiO_2_, thermal treatment is applied to TiO_2_ in the presence of a reducing gas (H_2_), to improve electrical conductivity.^43,49,50^ In the process, oxygen vacancies (O_V_) form in the TiO_2_ lattice, providing numerous anchoring sites for precious-metal catalysts, thereby enabling their high distribution. Studies also suggest that O_V_ also plays a role in forming M–OH_ads_ species, which promotes the bifunctional mechanism and enhances the electrocatalytic reaction.^32,51^ For instance, Lu et al.,^32^ reported the enhanced catalytic activity of Pd supported on hydrogen-treated TiO_2_ nanobelts (Pd/h-TiO_2_) relative to the untreated nanobelts (Pd/TiO_2_) and Pd/C. The authors confirmed enhanced catalytic activity for EOR owing to increased O_V_, identified by X-ray photoelectron spectroscopy (XPS) and electron paramagnetic resonance (EPR). The optimum activity of the Pd/h-TiO_2_ was attributed to the O_V_-rich support, which facilitated the oxidative removal of the reaction intermediate species like CH_3_CO_ads_ to acetate (CH_3_COO^–^). Naik et al.,^52^ demonstrated the preparation of oxygen-deficient black TiO_2_ nanosheets (B-TiO_2_-*x* NSs) using 1-butanol and successfully loaded Pd nanoparticles. The catalysts were applied to the glycerol oxidation reaction (GOR) and oxygen reduction reaction (ORR). The chronoamperometric (CA) measurements showed excellent stability of the Pd nanoparticles by exhibiting a slower rate of current decay relative to that of Pd/C, which was attributed to the SMSI between the Pd nanoparticles and reduced support. Another common strategy for preparing substoichiometric titanium includes doping TiO_2_ with other transition metals in the presence of H_2_ gas.^53–55^ For instance, in a study by Black-Araujo,^56^ the authors prepared silicon and molybdenum-doped titanium oxide (TOMS) as supports for Pt nanoparticles. They evaluated their behavior toward EORs in an alkaline electrolyte. Compared to Pt/C, Pt/TOMS exhibited a small charge transfer resistance (*R*_ct_) over temperatures of 10–50 °C and low activation energies, demonstrating the advantages of doped TiO_2_ (substoichiometric titanium). CeO_2_ has been widely studied as a promoter for supported precious-metal catalysts in electrocatalytic reactions such as EOR and ORR.^36,37,57,58^ In the EOR, the low redox potential between Ce^4+^ and Ce^3+^ improves the electrocatalytic reaction by facilitating the production of the O_V_^’^. This occurs through the reduction of Ce^4+^ (by accepting electrons) to form Ce^3+^, which is associated with the presence of O_V_ in the material, and as aforementioned, these O_V_’s are vital in the EOR for improved reaction kinetics and catalyst stability.^59–61^ Bifunctional Pd–CeO_2_ nanorods/C (Pd–CeO_2‑NR_/C) were studied toward oxygen reduction and ethanol oxidation reactions in alkaline electrolytes.^36^ The prepared catalyst Pd–CeO_2‑NR_/C exhibited a lower onset potential and higher mass and specific activities compared to Pd/C in both reactions. Furthermore, 98% of the ECSA was retained by the bifunctional catalyst relative to Pd/C, which shows 50% retention, demonstrating the improved stability of the catalyst Pd–CeO_2‑NR_/C.^36^ The roles of the M–OH_ads_ species and O_V_ in hydrated ceria (CeO_2_·*x*H_2_O) were investigated by Li and co-workers in Pd-catalyzed EORs.^62^ Based on the authors’ findings, both the O_V_ and M–OH_ads_ play significant roles in enhancing the stability and electrocatalytic activity of the Pd nanoparticles toward the reactions. Moreover, using CO stripping and density functional theory (DFT) calculations, the authors revealed that the downshifted ε_d_ in Pd led to weaker adsorption of the reaction intermediate species, promoting enhanced reactions.

Besides, the use of electrocatalysts developed for EORs in DEFCs is not limited to DEFCs. It can be applied in the electrochemical detection of ethanol from beverages,^63–65^ medicine,^66^ and biological samples,^67–69^ for quality control, clinical, forensics and road safety management purposes.^66–68^ Unlike chromatography,^70^ spectrometry,^65^ semiconductor,^71,72^ and enzymatic-based ethanol detection methods,^68^ the nonenzymatic amperometric sensor is attractive since it is portable, operates at low temperatures, and over a wide pH range.^66,73^ It is inexpensive and easy to operate, while providing rapid ethanol detection using low sample volumes.^73,74^ In this regard, exploring the multifunctional properties of the developed electrocatalyst in areas such as nonenzymatic amperometric sensors is worthwhile. Despite extensive studies on TiO_2_ and CeO_2_ as supports for precious-metal-based catalysts in ethanol oxidation reactions, there is a lack of research on heterostructured TiO_2_–CeO_2_ for EOR. At most, TiO_2_–CeO_2_ heterostructures have been explored for catalytic reactions involving CO, volatile organic compounds (VOC), biomass-derived oxidation, among others.^75–80^ Owing to the intrinsic properties shown by the TiO_2_–CeO_2_ phases, such as the rich O_V_, SMSI, and stability of the supported catalysts, it is worthwhile to investigate their behavior toward EOR in alkaline electrolyte.

Therefore, a sol–gel method followed by thermal treatment of TiO_2_, in the absence and presence of cerium under a reducing atmosphere, was used to prepare metal-oxide-based supports (TiO_2_ and Ce_2_Ti_2_O_7_–TiO_2_). A simple chemical (alcohol) reduction method was used to load Pd (10 wt %) nanoparticles to obtain the catalysts Pd/TiO_2_ and Pd/Ce_2_Ti_2_O_7_–TiO_2_. Unlike strong reducing agents such as sodium borohydride or sodium hydrazine, ethanol serves as both the reducing agent and solvent in this method, providing mild reaction conditions that are beneficial for the controlled synthesis of uniform, small-sized nanoparticles under monitored conditions.^81^ Raman spectroscopy, powder X-ray diffraction (PXRD), and transmission electron microscopy (TEM) demonstrated the successful formation of the Ce–Ti–O interface following the thermal treatment of TiO_2_–Ce. XPS analysis confirmed the presence of high concentrations of O_V_, M–OH_ads_ species, and SMSI on Pd/TiO_2_ and Pd/Ce_2_Ti_2_O_7_–TiO_2_. The Pd/Ce_2_Ti_2_O_7_ catalyst displayed enhanced reaction kinetics (Tafel slope 137.5 mV dec^–1^) and mass activity (1065 mA mg_Pd_^–1^) superior to Pd/TiO_2_ and Pd/C. The impressive electrocatalytic activity demonstrated by the catalyst was attributed to synergistic effects between Ce_2_Ti_2_O_7_–TiO_2_ and Pd nanoparticles, which resulted in a 3d–2p–4f orbital hybridization as shown by the DFT studies. This 3d–2p–4f orbital hybridization endowed the catalyst with fast electron transport and high tolerance to poisoning due to the downshifted ε_d_ of Pd.

## 2 Experimental Section

### 2.1 Materials and Reagents

Acetylene carbon black (TIMICAL SUPER CB, 45 m^2^ g^–1^) was acquired from Gelon, China. Titanium dioxide anatase (TiO_2_-A) was purchased from PAL chemicals. Cerium nitrate hexahydrate (Ce­(NO_3_)_3_·6H_2_O, 99.99%), Pluronic F127, potassium chloride (KCl, 99.0%), tetrachloropalladate (K_2_PdCl_4_, 99.99%), and Nafion perfluorinated resin solution (5.0 wt % mixture in lower aliphatic alcohols) were purchased from Sigma-Aldrich, South Africa. Ethanol (C_2_H_5_OH, 99.0%) and isopropanol (C_3_H_8_O, 99.0%) were acquired from MK chemicals. Ammonia solution (NH_4_OH, 25.0%) and potassium hydroxide pellets (KOH, ≥98.0%) were purchased from ACE chemicals. Nitrogen (N_2_), hydrogen (H_2_), and carbon monoxide (CO) gases were supplied by Afrox (South Africa). The ultrapure water was collected from a Millipore Milli-Q system with a resistivity of 18.2 MΩ cm.

### 2.2 Preparation of the TiO_2_-Based Supports

A previously described method was followed with slight modifications.^82^ The TiO_2_-based supports were prepared by dispersing 3 mmoles of TiO_2_-A in a mixture of ultrapure water and ethanol (30:70 vol %). Then, 2.0 wt % of Pluronic F127 surfactant was added to the mixture and dispersed by ultrasonication for 10 min. This suspension was left to stir for 3 h at ambient temperature. In the case of cerium titanate solid solution, Ce­(NO_3_)_3_·6H_2_O (1.0 mmol; 434.2 mg) was added to the dispersed TiO_2_-A suspension, followed by pH adjustment to 9.0 using NH_4_OH. After stirring for 5 h, the solvent was evaporated, and the sample was further dried at 80 °C for 12 h in a vacuum oven. The obtained powders were ground into fine powder and subjected to 850 °C heat treatment for 8 h under H_2_ and N_2_ (10:90 vol %) using a 5 °C min^–1^ ramping time. **CAUTION!** We declare here that no uncommon hazards are noted during the experimentation. However, caution is advised wherein every chemical reagent should be treated as poison which must never be ingested, and synthesis procedures carried out in a fume hood.

### 2.3 Preparation of the Catalysts

A chemical (ethanol) reduction method was used to prepare the catalysts, following a method previously reported by our group with slight modifications.^83^ The prepared supports (270.0 mg) were dispersed in 15.0 mL of water by ultrasonication for 30 min. Then, K_2_PdCl_4_ (28.19 mM; 10.0 mL) was added to the stirring support mixture. The pH was adjusted to 11.0, using 2.50 M KOH and followed by the addition of 25.0 mL of ethanol. The mixture was refluxed at 80 °C for an hour, cooled to ambient temperature, and centrifugally washed with water until the supernatant reached a neutral pH. Finally, the obtained catalysts were dried at 60 °C for 12 h in a vacuum oven.

### 2.4 Physical Characterization

Powder X-ray diffraction was used to determine the crystalline structures of the composite supports and respective catalysts. The PXRD patterns were recorded on a Bruker D2 Phaser powder X-ray diffractometer with a copper (Cu Kα, λ = 0.154060 nm) radiation X-ray source with operating conditions of 30 kV, a current of 10 mA over 2θ = 10–90° and a step size of 0.026°. The samples were ground into a fine powder using a mortar and pestle, loaded onto a sample holder, and flattened with a glass plate. The PXRD patterns were identified using the Inorganic Crystal Structure Database (ICSD). The samples were analyzed on a Thermo Scientific Smart Raman DXR2 using a 532 nm laser, with 25 μm gratings. A power of 5 mW was used, and the scan range was from 100 to 3500 cm^–1^. Ten background scans were collected, as well as 10 scans for each sample. Scanning electron microscopy (SEM) images were acquired using a Zeiss Crossbeam 540 operated at 2 kV for imaging and 20 kV for energy-dispersive X-ray (EDX) analysis. The microstructure of the catalysts was examined using high-resolution transmission electron microscopy (HR-TEM). The JEOL JEM-2100 at 200 kV HR-TEM and a Thermo Fisher EDX detector were used. The samples were dissolved in ethanol and sonicated for 30 min, then dispersed onto a carbon-coated copper grid and left to dry. XPS was used to evaluate the elemental surface compositions and oxidation states of the samples. The XPS analysis was conducted using a Thermo ESCA Lab 250Xi spectrometer with monochromatic Al Kα (1486.7 eV).

### 2.5 Electrochemical Characterization

Electrochemical experiments were conducted in a standard three-electrode cell. The cell consisted of the working electrode (glassy carbon electrode (GCE) 3 mm and 0.0707 cm^2^), a silver–silver chloride filled with 3.0 M KCl electrolyte as a reference electrode (Ag|AgCl (3.0 M KCl)), and a glassy carbon rod as a counter electrode connected to a Bio-Logic VMP 300 Potentiostat and operated on the EC-LAB software. All potentials in this work are referenced to a silver/silver chloride (Ag/AgCl, 3.0 M KCl). The electrochemical measurements were conducted using a clean GCE. Alumina slurry was used to polish the GCE, followed by rinsing with water, methanol, and wiping. The catalyst inks were prepared by dispersing 3.0 mg of the catalyst powder in 300 μL of a mixture of ultrapure water and isopropanol (30.0:70.0 vol %) and 20.0 μL of 5.0 wt % Nafion solution followed by sonication for 30 min. Then, 2.5 μL of the sonicated ink was pipetted onto a clean and dried surface at ambient temperature. Electrochemical measurements were conducted in N_2_-purged 0.5 M KOH, and the cyclic voltammogram (CV) was collected from −1.0 to 0.40 V at a scan rate (ν) of 50 mV s^–1^, to determine the electrochemically active surface area (ECSA) of the prepared catalysts. Carbon monoxide (CO) stripping measurements were conducted in a 0.5 M KOH electrolyte. **Caution!** CO is a toxic gas and must be used with caution. The CO measurements were conducted inside a fume hood. First, the electrolyte was saturated with N_2_ for 15 min, then saturated with CO for 20 min while the electrode potential was held at −0.10 V. Finally, N_2_ was purged into the electrolyte for 10 min to remove excess CO, and the CO stripping was recorded using CV from −1.0 to 0.40 V at a ν of 20 mV s^–1^.^36^ The behavior of the catalysts toward the ethanol oxidation reaction was examined in 0.5 M KOH containing 0.5 M ethanol (0.5 M KOH + 0.5 M EtOH) using a CV within a potential window ranging from −1.0 to 0.40 V at a scan rate of 50 mV s^–1^. The linear sweep voltammograms (LSV) were conducted in 0.5 M KOH + 0.5 M EtOH at 10 mV s_._^–1^ The scan rate study was conducted using CVs at various scan rates (25–300 mV s^–1^) in 0.5 M KOH + 0.5 M EtOH. The CA measurements were conducted in 0.5 M KOH + 0.5 M EtOH at −0.30 V. Electrochemical impedance spectroscopy (EIS) was performed in 0.5 M KOH + 0.5 M EtOH at −0.30 V, using a frequency ranging from 10.0 mHz to 500.0 Hz. The electrochemical detection of ethanol was conducted using a dip in solution Metrohm screen-printed electrode (C110) comprising a working (carbon), reference (silver), and auxiliary (carbon) electrode. All experiments were conducted at room temperature.

### 2.6 Density Functional Theory (DFT) Simulations Methodology

The Density Functional Theory (DFT) was implemented in BIOVIA Materials Studio to complement the experimental results. To achieve this, two BIOVIA Materials Studio modules were employed, the adsorption locator tools and DMol^3^. The adsorption locator tools were used to adsorb the CH_3_CO* adsorbate onto the surfaces of Pd, Pd/TiO_2_, and Pd/Ce_2_Ti_2_O_7_–TiO_2_, the atomic positions of which were obtained from the Materials Project database. The surfaces were set as Pd(1 1 1), Pd(1 1 1) TiO_2_(1 0 1) and Pd(1 1 1) Ce_2_Ti_2_O_7_(2̅ 1 2) TiO_2_(1 0 1), respectively. The choice of these surfaces was informed by the experimental results obtained from the PXRD data. The above structures were modeled into 3 × 3 supercells and thereafter optimized. Each of the optimized structures had the above adsorbates adsorbed separately. The adsorption distance for the adsorbates from the topmost atoms was set to 5 Å, which was the maximum acceptable (software-dependent) distance for the simulation to run, and the accuracy was set at 10^–6^ kcal mol^–1^. The accuracy threshold was set at 1 × 10^–6^ eV, and Perdew–Wang generalized-gradient approximation (PW91) functionals were employed. These functionals are known to yield more reliable values for the predicted energies and minimize the additional computational time.^84^ Once convergences were achieved, electronic spectroscopies, binding energies, and other electronic properties were obtained.

## 3 Results and Discussion

### 3.1 Physical Characterization

Figure 1a illustrates the preparation of the catalysts, and the detailed procedure followed is provided in the Experimental Section. First, the supports, titanium dioxide, and the titanium–ceria solid solution were prepared by a sol–gel method followed by annealing at 850 °C under the reducing atmosphere comprising N_2_/H_2_ (90:10 vol %). Then, the Pd nanoparticles were grown on the supports, yielding Pd/TiO_2_ and Pd/Ce_2_Ti_2_O_7_–TiO_2_. Figure 1b compares the powder X-ray diffraction (PXRD) (i) TiO_2_, (ii) Pd/TiO_2_, (iii) Ce_2_Ti_2_O_7_–TiO_2_, and (iv) Pd/Ce_2_Ti_2_O_7_–TiO_2_, while Figure 1c represents the expanded section of the 2θ = 25–26° region of the supports and catalysts. The PXRD pattern of the TiO_2_-A (the precursor) is shown in the Supporting Information, Figure S1, exhibiting narrow peaks due to the high crystallinity of the titania anatase. The Miller indices (1 0 1), (0 0 4), (2 0 0), (1 0 5), (2 1 1), (2 0 4), (1 1 5), (2 2 0) and (2 1 5) reflecting at 2θ = 25.5, 37.8, 48.2, 53.9, 55.4, 62.8, 68.8, 70.5, and 75.2° are indexed to the tetragonal anatase phase (PDF 021-1272).^85^ The observation of the Miller indices corresponding to the tetragonal anatase phase in Figure 1b is noteworthy, as they indicate its retention after thermal treatment. The diffraction pattern of TiO_2_ (Figure 1b,i) exhibited an additional peak at 2θ = 27.5° indexed to the rutile phase (PDF 021-1276), which indicated that a smaller degree of nucleation occurred upon the subjection of TiO_2_-A to high temperatures (>400 °C).^86^ In contrast to TiO_2_, the presence of ceria in TiO_2_ inhibited the phase transformation of TiO_2_ and indicated the formation of the Ce–O–Ti interface (Figure 1b,iii), as demonstrated in literature.^44,87^ Indeed, the PXRD pattern of Ce_2_Ti_2_O_7_–TiO_2_, confirmed the existence of new peaks ascribed to cerium titanate (Ce_2_Ti_2_O_7_) PDF 047-0667.^88^ The inspection of the (101) diffraction peaks, as shown in Figure 1c demonstrated that the presence of Pd on TiO_2_ and Ce_2_Ti_2_O_7_–TiO_2_ leads to negative shifts in the Bragg angle and is attributed to lattice expansion of TiO_2_. On the other hand, the corresponding catalysts Pd/TiO_2_ and Pd/Ce_2_Ti_2_O_7_–TiO_2_ (Figure 1b,ii and 1b,iv) exhibited face-centered cubic (fcc) phases attributed to (111), (200), and (220) of the Pd (PDF: 01-087-0638). The average crystallite sizes (*D*) of the Pd nanoparticles were estimated using Scherrer’s equation (eq S1), and the determined *D* were 3.81 and 5.74 nm for Pd/TiO_2_ and Pd/Ce_2_Ti_2_O_7_–TiO_2_, respectively.

**Figure 1:**
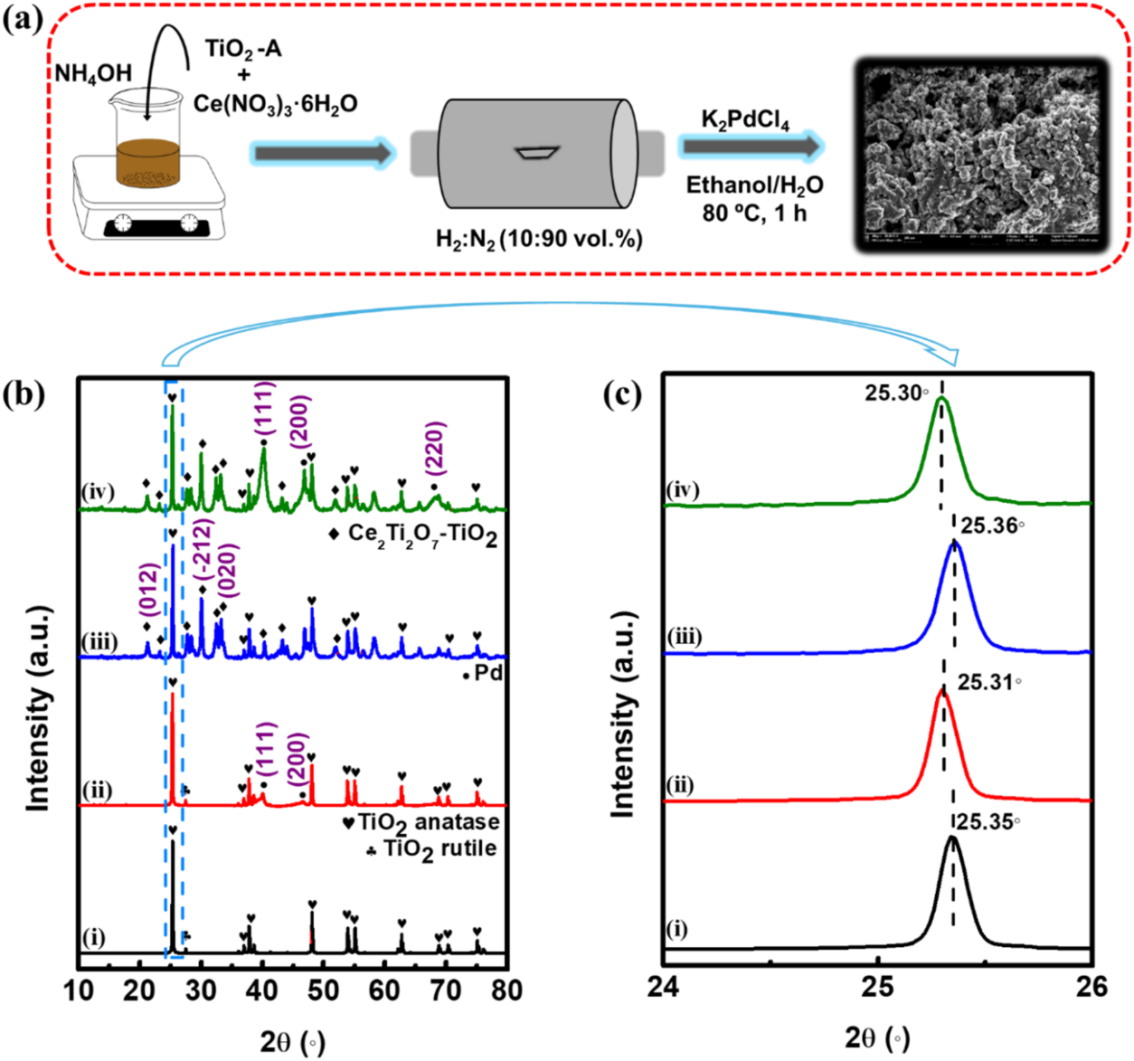
(a) The preparation of the supports and the catalysts, (b) PXRD patterns of (i) TiO_2_, (ii) Pd/TiO_2_, (iii) Ce_2_Ti_2_O_7_–TiO_2_, and (iv) Pd/Ce_2_Ti_2_O_7_–TiO_2_, and (c) the expanded portion of the Miller index (101) peak pattern of (b) for titania in TiO_2_, Pd/TiO_2_, Ce_2_Ti_2_O_7_–TiO_2_, and Pd/Ce_2_Ti_2_O_7_–TiO_2_.

The Raman spectra for TiO_2_-A, TiO_2_, and Ce_2_Ti_2_O_7_–TiO_2_ (Supporting Information, Figure S2) exhibit the characteristic peaks of TiO_2_ anatase comprising the E_g_, B_1g_ and A_1g_ vibrational modes at 196.6 cm^–1^, 399.6 cm^–1^, 515.8 cm^–1^ (B_1g_), and 644.2 cm^–1^ (A_1g_), respectively. A closer examination of the intense vibration mode E_g_ located at 142.1 cm^–1^ for TiO_2_-A was conducted after thermal treatment of the supports; TiO_2_ (142.2 cm^–1^) and Ce_2_Ti_2_O_7_–TiO_2_ (143.5 cm^–1^) exhibit an upward shift in wavenumber. The formation of the Ce–Ti–O interface resulted in more structural modifications relative to TiO_2_-A. Also, the absence of vibrational modes ascribed to CeO_2_ suggests that CeO_2_ is not the dominant phase in the material, which supports the PXRD observations and the successful formation of the cerium titanate solid solution under the applied conditions. The existence of oxygen vacancies in such materials has been linked to peak broadening and upward shifts. Based on the results obtained, it suggests that the presence of ceria favored the production of more O_V_ compared to the thermal treatment of TiO_2_-A.^85,89^

The morphology and elemental composition of the catalysts were probed using an SEM image and SEM-EDX. Figure S3 shows the SEM images and the corresponding EDX spectra for Pd/TiO_2_ and Pd/Ce_2_Ti_2_O_7_–TiO_2_. Both materials consist of granular-like particles. Pd/TiO_2_ (Figure S3a) depicts smooth surfaces with small spherical nanoparticles, while Pd/Ce_2_Ti_2_O_7_–TiO_2_ (Figure S3b) consists of aggregated small nanoparticles. The EDX (Figure S3c,d) clearly confirms the existence of the expected elemental compositions (i.e., Pd, titanium (Ti), Ce, and oxygen (O)).

The crystallite structure and morphology of the catalysts were further confirmed by using TEM and HR-TEM (Figure 2). As in the SEM images, Pd/TiO_2_ (Figure 2a) comprises smooth, spherical TiO_2_ particles with well-distributed Pd nanoparticles supported over them. In contrast, cerium titanate (Figure 2f) depicts a morphology distinct from that of the TiO_2_ due to the generation of the Ce–Ti–O interface in the Ce_2_Ti_2_O_7_–TiO_2_ support. The interplanar spacings of anatase TiO_2_(1 0 1), Ce_2_Ti_2_O_7_(2̅ 1 2), and Pd(1 1 1) were determined from the HR-TEM images and shown in Figure 2b–d and 2g–i for Pd/TiO_2_ and Pd/Ce_2_Ti_2_O_7_, respectively. Both catalysts revealed interplanar distances of ca. 0.350 and 0.230 nm for TiO_2_(1 0 1) and Pd(1 1 1), respectively, which is consistent with the literature.^49,87^ Similarly, Ce_2_Ti_2_O_7_(1 1 2) with an interplanar spacing of 0.297 nm confirmed the formation of the cerium titanate.^88^ The histogram in Figure S4 shows the Pd nanoparticles’ size distribution on both catalysts, ranging from 3.44–5.99 to 3.57–5.74 nm. Notably, the average crystallite sizes of Pd nanoparticles on Pd/TiO_2_ and Pd/Ce_2_Ti_2_O_7_–TiO_2_ were 4.77 and 5.09 nm, respectively, which corroborated the XRD data. Figure S5 shows high-angle annular dark field scanning transmission electron microscopy (HAADF-STEM) with the EDX elemental mapping for Pd/TiO_2_. The elements Pd, Ti, and O were present and uniformly distributed across the sample. Similarly, the HAADF-STEM with the EDX elemental mapping of Pd/Ce_2_Ti_2_O_7_–TiO_2_ is shown in Figure 2j–n, which confirms the presence of Pd, Ti, O, and Ce and their uniform distribution across the sample. More importantly, the Ti, Ce, and O show uniform coverage on the Ce–Ti–O interphase.

**Figure 2:**
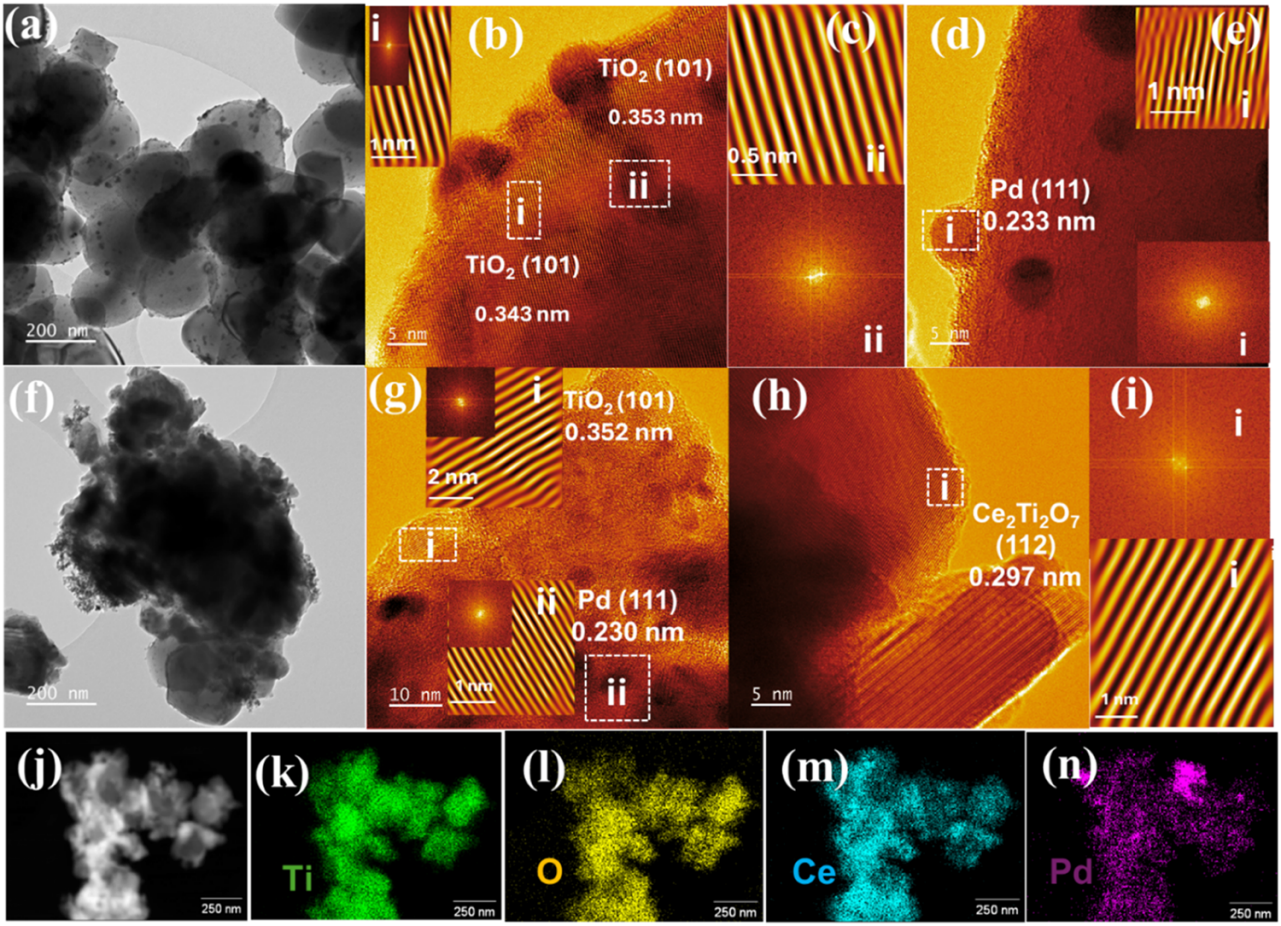
TEM image of (a) Pd/TiO_2_, (b–e) HR-TEM lattice parameters of TiO_2_ and Pd extracted by Fourier transform; (f) TEM image of Pd/Ce_2_Ti_2_O_7_–TiO_2_, (g–i) HR-TEM lattice parameters of TiO_2_, Pd, and Ce_2_Ti_2_O_7_ extracted by Fourier transform; (j) HAADF-STEM and (k–n) EDS elemental mapping of Pd/Ce_2_Ti_2_O_7_–TiO_2_.

XPS data provide important information about the catalysts’ chemical composition, valence states, and chemical bonding. The XPS survey spectra for TiO_2_-A, Pd/TiO_2_, and Pd/Ce_2_Ti_2_O_7_–TiO_2_ (Figure S6) show the presence of the expected chemical compositions, and the core level spectra of Ti 2p and O 1s for TiO_2_-A are shown in Figure S7. The Ti 2p spectrum (Figure S7a) revealed two distinct peaks corresponding to Ti 2p_1/2_ (463.59 eV) and Ti 2p_3/2_ (457.7 eV) attributed to Ti^4+^ species in the crystal lattice. On the other hand, the O 1s spectrum (Figure S7b) shows three prominent peaks, ascribed to the oxygen lattice (O_L_), O_V_, and the M–OH at binding energies of 529.0, 530.14, and 531.5 eV, respectively, which is in agreement with the literature.^90^

The Ti 2p, O 1s, Pd 3d, and Ce 3d of the catalysts Pd/TiO_2_ and Pd/Ce_2_Ti_2_O_7_–TiO_2_ are presented in Figure 3. The TiO_2_-A (Figure S7a) exhibited the Ti 2p_3/2_ peak at 457.7 eV, while positive shifts in binding energies were observed for Pd/TiO_2_ (458.8 eV) and Pd/Ce_2_Ti_2_O_7_–TiO_2_ (458.3 eV). This indicated the change in the local environment of Ti^4+^ attributed to an electron transfer from the support to the Pd nanoparticles, which has a significant influence on the SMSI between the Pd and the supports.^46,59,82^ It simply means that the introduction of ceria titanate onto TiO_2_ leads to electron-rich Pd/Ce_2_Ti_2_O_7_–TiO_2_, aided by the multiorbital (p–d–f) interactions arising from the hybridization between the Ti 2p, Pd 3d, and Ce 4f. This study suggests a novel perspective for constructing a unique electronic modulation strategy involving 2p–3d–4f orbital coupling. As would be seen later, this finding was further interrogated using DFT calculations.

**Figure 3:**
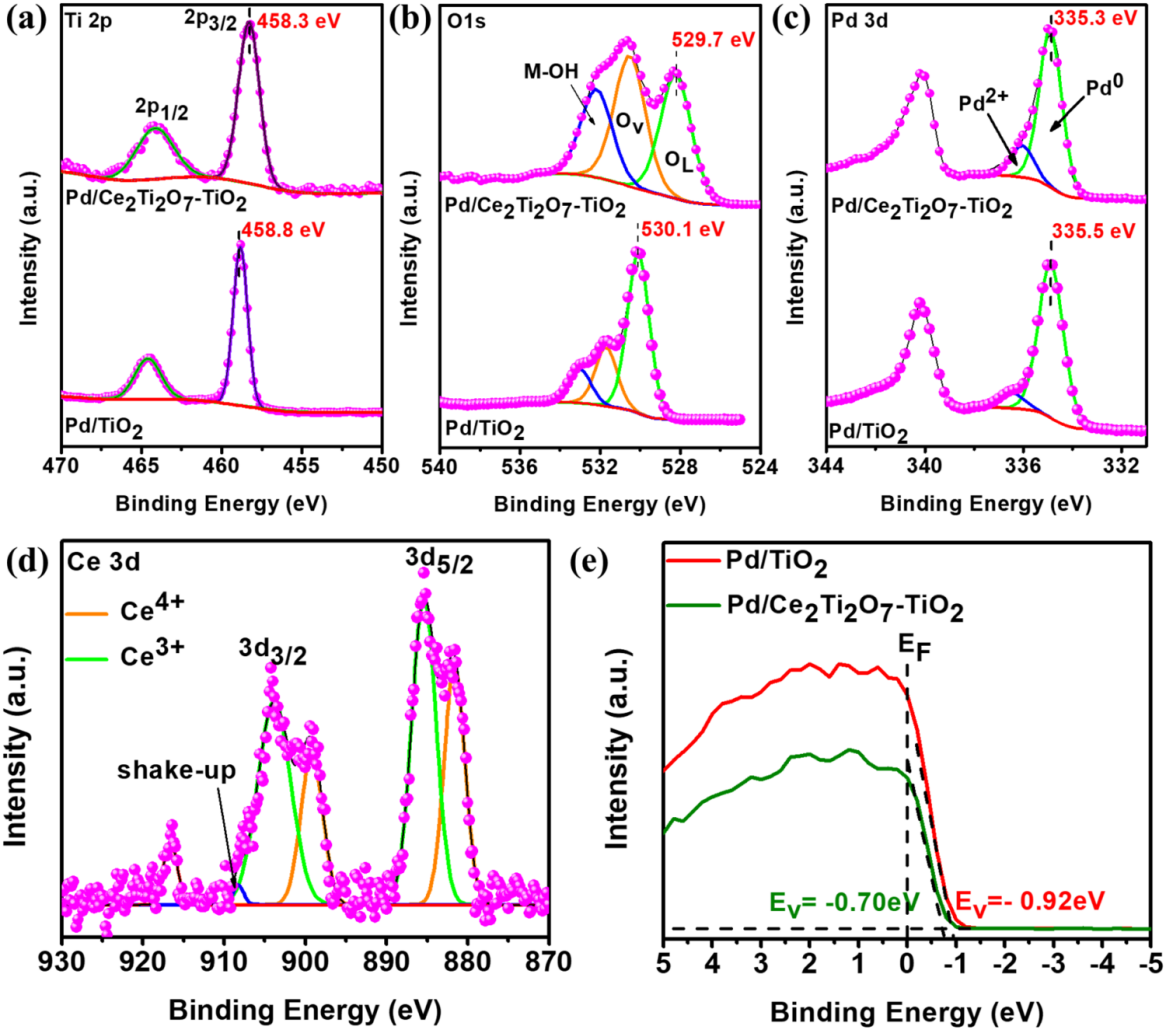
XPS core level spectra of Pd/TiO_2_ and Pd/Ce_2_Ti_2_O_7_–TiO_2_, (a) Ti 2p, (b) O 1s, (c) Pd 3d, (d) Ce 3d, and (e) valence band spectra of Pd/TiO_2_ and Pd/Ce_2_Ti_2_O_7_–TiO_2_.

Also, the deconvoluted O 1s spectra of the catalysts (Figure 3b) exhibit three oxygen species: O_L_ describing the surface lattice oxygen (i.e., metal–oxygen bonds), O_V_ representing the defect sites/surface oxygen species from low coordinated oxygen atoms or vacancies, while M–OH_ads_ is due to surface-adsorbed oxygen species such as water molecules. The determined percentage concentrations of O_V_, M–OH, and O_L_ are 37.2, 37.3 and 25.6% for Pd/Ce_2_Ti_2_O_7_–TiO_2_, and 23.3, 28.4, and 48.4% for Pd/TiO_2_, respectively. Pd/Ce_2_Ti_2_O_7_–TiO_2_ exhibited a higher content of M–OH, which is beneficial for the bifunctional mechanism. Moreover, the O_V_/O_L_ (peak area) ratio showed a higher O_V_ content in Pd/Ce_2_Ti_2_O_7_–TiO_2_ (1.45) relative to Pd/TiO_2_ (0.48), further demonstrating that cerium titanate strongly favored the formation of O_V_ and supporting the findings from Raman analysis. It is well documented that O_V_ facilitates the production of M–OH_ads_ species, which promotes the bifunctional mechanism and allows for an efficient charge transport to be realized.^32,51,85^ Hence, it can be suggested that Pd/Ce_2_Ti_2_O_7_–TiO_2_ will exhibit improved electrocatalytic performance toward EOR in comparison to Pd/TiO_2_ due to higher promotion of the removal of reaction intermediate species and enhanced electron transfer properties.

Figure 3c depicts the Pd 3d spectra with two peaks located at binding energies of ca. 340.0 and 335.0 eV ascribed to Pd 3d_3/2_ and Pd 3d_5/2_. The deconvolution of Pd 3d_5/2_ resulted in a doublet ascribed to Pd^2+^ and Pd^0^. The Pd^0^ and Pd^2+^ peaks in Pd/C metal are located at binding energies of 335.5 and 336.8 eV, respectively.^63^ As can be seen in Figure 3c, slight negative shifts were observed for both Pd states on Pd/TiO_2_ (334.9; 336.5 eV) and Pd/Ce_2_Ti_2_O_7_–TiO_2_ (334.9; 336.0 eV), respectively. This result confirmed electron transfer from the support to Pd, indicating the presence of the SMSI.^43,63^ The presence of Pd^2+^ indicated interfacial interactions between the Pd nanoparticles and the support in both catalysts. In contrast to Pd/TiO_2_, Pd/Ce_2_Ti_2_O_7_–TiO_2_ revealed a higher Pd^2+^ ratio_,_ suggesting that more interfacial interactions exist in the latter catalyst. Lastly, the Ce 3d spectra are shown in Figure 3d, which display two regions of Ce 3d_5/2_ and Ce 3d_3/2_. After deconvolution, the Ce 3d_3/2_ and Ce 3d_5/2_ exhibit four peaks at binding energies of 881.0, 899.20, 916.6 eV and 885.4, 903.8 eV, which were obtained and attributed to Ce^4+^ and Ce^3+^, respectively.^91^ A higher concentration of Ce^3+^ (60.6%) relative to Ce^4+^ (39.4%) was found on Pd/Ce_2_Ti_2_O_7_–TiO_2_, further corroborating the abundant O_V_ present in the Ce_2_Ti_2_O_7_–TiO_2_ support. Notably, the atomic ratio of Pd to Ti in TiO_2_ and Ce_2_Ti_2_O_7_–TiO_2_ was determined as 0.35 and 0.78, respectively. This result implies that O_V_-rich Ce_2_Ti_2_O_7_–TiO_2_ support provided a higher loading of Pd, by exposing an abundance of anchoring sites for Pd nanoparticles.^83,85^

Furthermore, XPS was used to gain insight into the electronic structures of Pd/TiO_2_ and Pd/Ce_2_Ti_2_O_7_–TiO_2_. The valence band maxima (*E*_v_) of Pd/TiO_2_ and Pd/Ce_2_Ti_2_O_7_–TiO_2_ were acquired from the valence band spectra and estimated to be −0.92 and −0.70 eV, respectively (Figure 3e). Since the *E*_v_ represents the highest occupied molecular orbital (HOMO), the acquired values indicated that the HOMO of Pd/Ce_2_Ti_2_O_7_–TiO_2_ shifted to a higher energy level compared to Pd/TiO_2_.^92,93^ In other words, the *E*_v_ value of the Pd/Ce_2_Ti_2_O_7_–TiO_2_ is closer to the Fermi energy and thus easier for electrons to travel to the conduction band than that for the Pd/TiO_2_.

### 3.2 Electrochemical Characterization

#### 3.2.1 Electro-Oxidation of Ethanol

The electrocatalytic activities of the prepared materials, Pd/TiO_2_ and Pd/Ce_2_Ti_2_O_7_–TiO_2_, were evaluated toward electro-oxidation of ethanol using 0.5 M KOH as the supporting electrolyte, and an in-house prepared catalyst consisting of Pd supported on carbon black (Pd/C) with the same Pd weight (10 wt %) was used as a benchmark catalyst. Figure S8a depicts the CV of the catalysts in 0.5 M KOH saturated with N_2_ collected at a potential sweep rate of 50 mV s^–1^. All the catalysts exhibited the typical voltametric features of Pd-based catalysts, i.e., hydrogen desorption and adsorption peaks at −0.50 to −1.0 V vs Ag/AgCl and the Pd-oxide (PdO) reduction peak at −0.20 to −0.60 V vs Ag/AgCl, showing the presence of Pd in all materials.^91,94,95^ The integrated area under the PdO reduction peak was used to determine the electrochemically active surface area (ECSA) of the catalysts using eq S1. Based on the calculated values, the ECSA increased in the following order: Pd/C (22.36 cm^2^ mg_Pd_^–1^), Pd/TiO_2_ (236.7 cm^2^ mg_Pd_^–1^), and Pd/Ce_2_Ti_2_O_7_–TiO_2_ (343.2 cm^2^ mg_Pd_^–1^), demonstrating a higher ECSA for Pd/Ce_2_Ti_2_O_7_–TiO_2_ relative to Pd/TiO_2_ and Pd/C. Unlike TiO_2_, Ce_2_Ti_2_O_7_–TiO_2_ exhibited a high concentration of O_V_, which provided a high surface area for high distribution of the Pd nanoparticles.^55^ In the presence of 0.5 M EtOH (Figure 4a), two distinct anodic peaks were observed in the forward and reverse scans of the CV curves for all catalysts, demonstrating their activity toward the EOR. In the forward scan, oxidation of the freshly adsorbed ethanol molecules occurs, whereas the reverse peak is attributed to the removal of the adsorbed intermediates, leading to product formation.^66^ According to literature, ethanol oxidation in alkaline electrolyte solution undergoes the following steps (represented in eqs 1–4):^17^

1 $$\mathrm{Pd}+{\mathrm{CH}}_{3}{\mathrm{CH}}_{2}\mathrm{OH}↔\mathrm{Pd}\text{−}{\mathrm{CH}}_{3}{\mathrm{CH}}_{2}{\mathrm{OH}}_{\mathrm{ads}}$$

2 $$\mathrm{Pd}\text{−}{\mathrm{CH}}_{3}{\mathrm{CH}}_{2}{\mathrm{OH}}_{\mathrm{ads}}+3{\mathrm{OH}}^{-}\to \mathrm{Pd}\text{−}{\mathrm{CH}}_{3}{\mathrm{CO}}_{\mathrm{ads}}+3H_{2}O+3e^{-}$$

3 $$\mathrm{Pd}\text{−}{\mathrm{CH}}_{3}{\mathrm{CO}}_{\mathrm{ads}}+\mathrm{Pd}\text{−}{\mathrm{OH}}_{\mathrm{ads}}\to \mathrm{Pd}\text{−}{\mathrm{CH}}_{3}{\mathrm{COOH}}_{\mathrm{ads}}+\mathrm{Pd}\left(rds\right)$$

4 $$\mathrm{Pd}\text{−}{\mathrm{CH}}_{3}\mathrm{COOH}+{\mathrm{OH}}^{-}\to \mathrm{Pd}\text{−}{\mathrm{CH}}_{3}{\mathrm{COO}}^{-}+H_{2}O\left(\mathrm{fast}\right)$$

**Figure 4:**
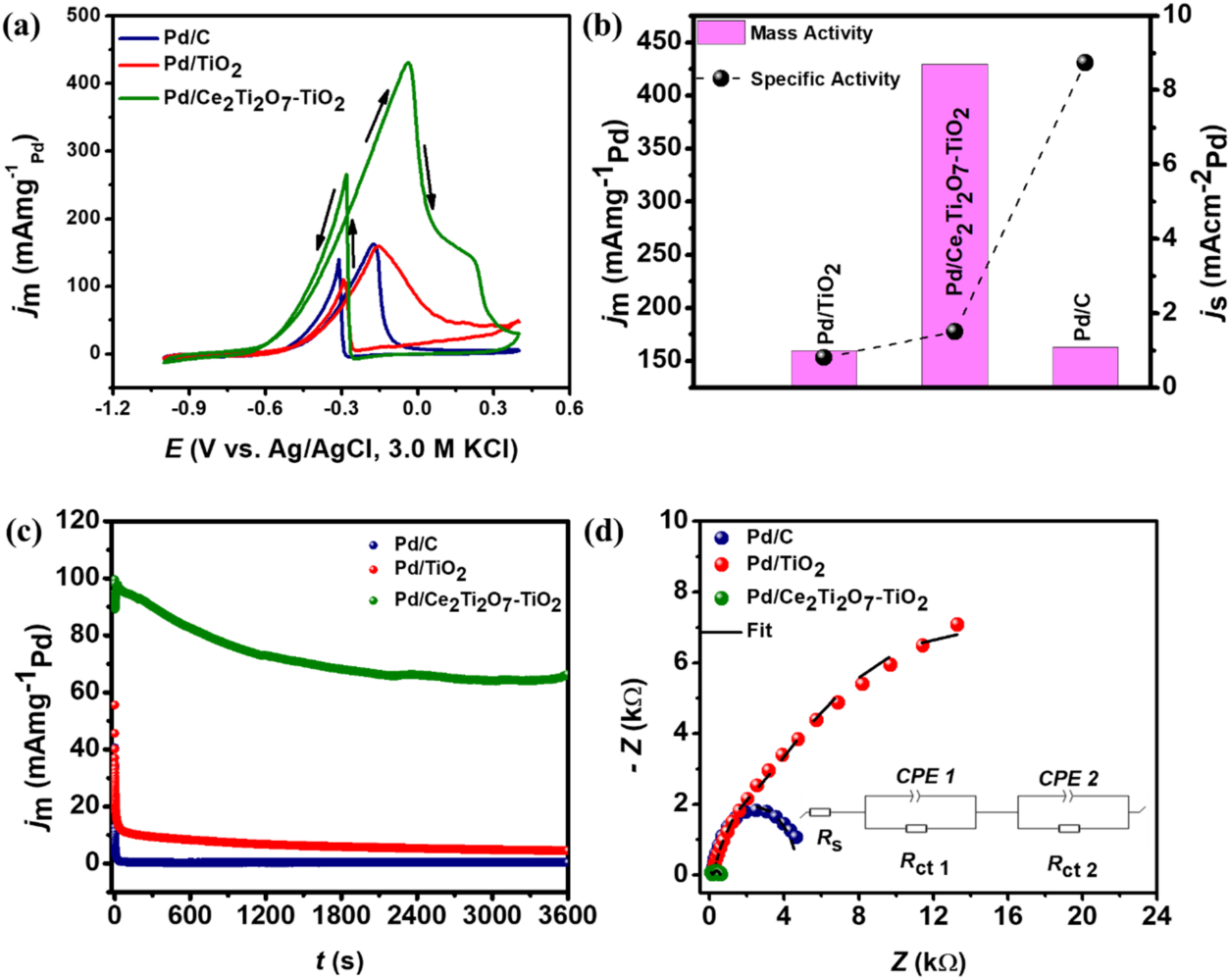
(a) CV profiles in 0.5 M KOH + 0.5 M EtOH collected at 50 mV s^–1^, (b) mass and specific activities bar graph, (c) EIS Nyquist plots, and (d) CA in 0.5 M KOH + 0.5 M EtOH collected at −0.30 V for Pd/TiO_2_, Pd/Ce_2_Ti_2_O_7_–TiO_2_, and Pd/C.

First, the ethanol molecules adsorb onto the Pd surface (eq 1) and are oxidized into CH_3_CO_ads_ species through a three-electron process (eq 2). Then, the adsorbed OH_ads_ on the adjacent Pd react with the CH_3_CO_ads_ species, forming adsorbed acetic acid (CH_3_COOH) as shown in eq 3. Finally, the CH_3_COOH reacts with the present OH^–^ ions, forming the CH_3_COO^–^ ions, which desorb from the active sites, revealing more active sites for EOR (eq 4). Herein, it is postulated that the presence of promoters, TiO_2_ and Ce_2_Ti_2_O_7_–TiO_2_, with the abundant O_V_, will enhance the electrocatalytic reaction by facilitating the dissociation of water molecules (generating more M–OH_ads_) at earlier potentials for the bifunctional mechanism to occur, endowing the catalyst with more tolerance to poisoning by the intermediate reaction species.^40^ Therefore, carbon monoxide (CO) stripping tests were performed using CV in 0.5 M KOH saturated with CO, as shown in Figure S8b. Evidently, all three catalysts exhibited anodic peaks in the potential region of −300 to −84.5 mV due to the electro-oxidation of CO. The catalysts Pd/Ce_2_Ti_2_O_7_–TiO_2_ (−290 mV) and Pd/TiO_2_ (−283 mV) showed earlier onset potentials (*E*_0_'s) than Pd/C (−220 mV), as well as higher peak currents. These results confirm that in comparison to Pd/C, the Pd/Ce_2_Ti_2_O_7_–TiO_2_ and Pd/TiO_2_ catalysts promote the bifunctional mechanism (i.e., CO_ads_ + M–OH_ads_ → CO_2_ + H_2_O) and were characterized as being less susceptible to CO-poisoning.^96,97^ Moreover, in the EOR, the catalysts Pd/TiO_2_ (−596 mV) and Pd/Ce_2_Ti_2_O_7_–TiO_2_ (−700 mV) exhibited earlier *E*_0_’s, relative to Pd/C (−556 mV), which illustrates the reduced activation energy barriers to the reaction on the metal-oxide-based catalysts. Particularly, Pd/Ce_2_Ti_2_O_7_–TiO_2_, which exhibited 104 and 144 mV negative shifts in *E*_0_ compared to Pd/TiO_2_ and Pd/C, respectively. The same observations were realized by linear sweep voltammetry (LSV), which was conducted to further investigate the reaction kinetics at each electrode (Figure S9a). As in the CV profiles, all catalysts exhibited an anodic peak attributed to ethanol electro-oxidation, with Pd/Ce_2_Ti_2_O_7_–TiO_2_ exhibiting a lower *E*_0_ and a high current density (*j*). Additionally, the Tafel slope (*b*) values were obtained from the potential vs log *j* plot as defined in eqs S3, S4 and shown in Figure S9b. The acquired values were 172.2, 137.5, and 138.5 mV dec^–1^ for Pd/TiO_2_, Pd/Ce_2_Ti_2_O_7_–TiO_2_, and Pd/C, respectively, which implies that the reaction kinetics were accelerated on Pd/Ce_2_Ti_2_O_7_–TiO_2_, and Pd/C compared to Pd/TiO_2_. This accounts for the superior mass activity (*j*_m_) demonstrated by Pd/Ce_2_Ti_2_O_7_–TiO_2_ (0.423 A mg_Pd_^–1^), which was 2.70- and 2.63-fold relative to Pd/TiO_2_ (0.160 A mg_Pd_^–1^) and Pd/C (0.163 A mg_Pd_^–1^) as shown in Figure 4b. The enhanced catalytic behavior shown by Pd/Ce_2_Ti_2_O_7_–TiO_2_ was due to the O_V_-rich Ce_2_Ti_2_O_7_–TiO_2_, which promoted the bifunctional mechanism and improved electron transport.^41^ It also suggests that the metal–support interaction was significantly enhanced on the Pd/Ce_2_Ti_2_O_7_–TiO_2_ relative to Pd/TiO_2_, providing optimal adsorption and desorption of the adsorbates.^31^ Figure 4b also shows the specific activities (*j*_s_) of the catalysts, Pd/Ce_2_Ti_2_O_7_–TiO_2_ (1.50 mA cm^–2^), Pd/TiO_2_ (0.81 mA cm^–2^), and Pd/C (8.74 mA cm^–2^). Notably, Pd/C exhibited 10.8- and 5.8-fold *j*_s_ relative to Pd/TiO_2_ and Pd/Ce_2_Ti_2_O_7_–TiO_2_, respectively. It is commonly understood that specific activity relates to the performance per active site, while mass activity measures the overall performance of the catalyst based on its total mass. For a real-world application such as fuel cells (as in this study), mass activity is preferred over the specific activity due to cost implications; the cost of the precious-metal catalyst is tied to its quantity/mass.

The catalyst’s tolerance to poisoning was further probed using the chronoamperometric (CA) measurements (Figure 4c). As seen in the CA profile, within the first few seconds, all catalysts exhibit rapid decay in *j*_m_, due to the adsorption of ethanol and the reaction intermediates on the active sites.^18,98^ As time progresses, *j*_m_ reaches a steady state, and the rate at which it occurs correlates with the rate at which the catalyst is poisoned. Among the three catalysts, the CA profile of Pd/C showed high susceptibility to poisoning with a rapid decay in *j*_m_, while Pd/TiO_2_ and Pd/Ce_2_Ti_2_O_7_–TiO_2_ exhibited slower *j*_m_ decay, indicating their tolerance to poisoning. This was attributed to the oxophilic supports, which enhanced the bifunctional mechanism, particularly, Pd/Ce_2_Ti_2_O_7_–TiO_2_, which exhibited a significant retention of *j*_m_ at the end of the CA measurement.^24,40^ This result further corroborates the Raman and XPS analyses, which demonstrated O_V_-rich Ce_2_Ti_2_O_7_–TiO_2_ support that promoted the bifunctional mechanism and antipoisoning capabilities.^62^ To gain further insight into the surface properties of the catalysts, electrochemical impedance spectroscopy (EIS) was performed at a constant potential of −0.30 V. Figure 4d displays the Nyquist plots, and the inset shows the electrochemical equivalent circuit (EEC) used to fit the plots. The EEC comprises the following elements: the solution resistance (*R*_s_), the constant phase element (CPE) due to the pseudocapacitive behavior of the catalysts, and the charge transfer resistance (*R*_ct_), respectively.^99^ The values obtained from the fittings are reported in Table S1. Notably, Pd/Ce_2_Ti_2_O_7_–TiO_2_ showed a smaller total *R*_ct_ (0.553 kΩ) in comparison to Pd/TiO_2_ (27.12 kΩ) and Pd/C (4.79 kΩ), which was also represented by the small arcs depicted in Figure 4d. This result demonstrates that Pd/Ce_2_Ti_2_O_7_–TiO_2_ exhibited enhanced charge transfer at the electrode surface, due to improved electrode–electrolyte interfacial properties relative to Pd/TiO_2_ and Pd/C. It was also observed that the total CPE value for Pd/Ce_2_Ti_2_O_7_–TiO_2_ (120.6 μF s^*a*–1^) was higher than that of the Pd/TiO_2_ (82.7 μF s^*a*–1^) and Pd/C (57.8 μF s^*a*–1^), which was attributed to the surface roughness and heterogeneity as observed in the SEM and TEM images. Furthermore, the ideality factor (*a*) values obtained from the impedance due to the CPE defined in eq S2 were 0.613, 0.674, and 0.884 for Pd/TiO_2_, Pd/Ce_2_Ti_2_O_7_–TiO_2_, and Pd/C, respectively. All catalysts showed a pseudocapacitive (mixed adsorptive–diffusive) behavior, and the higher value observed for Pd/C was due to the nanoporous carbon black, which exhibits more of a capacitive behavior.^63,99,100^ Table 1 shows a comparison of our best-performing catalyst, Pd/Ce_2_Ti_2_O_7_–TiO_2_ (5.0 μg_Pd_) displayed in Figure S10, with other Pd-based catalysts reported in the literature. Notably, Pd/Ce_2_Ti_2_O_7_–TiO_2_ catalyst exhibited electrocatalytic behavior comparable to those reported in other studies, demonstrating its promising electrochemical performance toward EORs.

**Table 1:** Comparison of the EOR Performance of Pd/Ce_2_Ti_2_O_7_–TiO_2_ with Those of Other Pd-Based Catalysts

catalyst	*E*_onset_ (V)	*b* (mV dec^–1^)	mass activity (mA mg_Pd_^–1^)	refs
Pd/Ce_2_Ti_2_O_7_–TiO_2_	–0.70	137.5	1065	this work
Pd/NiWO_4_/C	–0.60		1510	^22^
Pd_10_(CeO_2_NR)_20_(Vn)_70_	–0.70	143.1	1780	^36^
Pd–CeO_2_/OLC	–0.68	145.0	1180	^63^
Pd–CeO_2‑NR_/C	–0.72		697.0	^101^
Pd NPs@Ni SAC			1093	^102^
Pd–NiCoO_*x*_/C	–0.56	179.5	430.0	^103^

The stability of the best-performing material, Pd/Ce_2_Ti_2_O_7_–TiO_2_, was compared to that of the Pd/C catalyst by sweeping 1000 cycles in 0.5 M KOH at a scan rate of 100 mV s^–1^ from −1.0 to 0.4 V (Figure S11a,b). Both catalysts showed an increase in ECSA over the first few cycles, reaching a maximum at the 500th cycle; thereafter, the ECSA decreased. As observed in Figure S11c, the Pd/Ce_2_Ti_2_O_7_–TiO_2_ catalyst retained a higher ECSA than Pd/C, showing a small loss of 18.8% in comparison to that of Pd/C with 33.2% ECSA loss. This result illustrates the improved stability of the Pd nanoparticles on the Ce_2_Ti_2_O_7_–TiO_2_ support, due to an enhanced interaction between the metal catalyst and the support, further suggesting that there was minor dissolution and aggregation of Pd nanoparticles on Pd/Ce_2_Ti_2_O_7_–TiO_2_ compared to Pd/C after the ADT.^42,104^

#### 3.2.2 DFT Calculations

The experimental results show that Pd/Ce_2_Ti_2_O_7_–TiO_2_ exhibits enhanced electrocatalytic activity for EOR in an alkaline electrolyte compared to that of Pd/TiO_2_ and Pd/C. Next, DFT calculations were performed to provide further insights into these experimental observations. Figure S12a–c shows the graphical presentation of the three catalyst surfaces modeled using the BIOVIA Materials Studio as described in the Experimental Section, as well as the proposed adsorption sites of the acetyl (CH_3_CO*) adsorbate. The choice of adsorbate was informed by previous studies demonstrating that the complete oxidation of ethanol (i.e., C–C bond cleavage in ethanol) does not occur instantaneously.^105–107^ According to the literature, ethanol molecules undergo dehydrogenation to form the CH_3_CO* species as shown in eqs 1 and 3. Subsequently, acetic acid (CH_3_COOH) can be formed with the available hydroxyl species (OH*), eq 4. The adsorbates’ adsorption energies were determined using eq 5:^105–107^

5 $$E_{\mathrm{ads}}=\left(E_{\mathrm{surface}+\mathrm{adsorbate}}\right)-\left(E_{\mathrm{surface}}+E_{\mathrm{adsorbate}}\right)$$

where *E*_ads_ is the adsorption energy, *E*_surface+adsorbate_ is the overall energy of the catalyst surface with the adsorbate, and *E*_surface_ + *E*_adsorbate_ is the sum of the catalyst surface and the adsorbate. The *E*_ads_ for CH_3_CO* on Pd, Pd/TiO_2_, and Pd/Ce_2_Ti_2_O_7_–TiO_2_ are −0.480, −0.503, and −0.433 eV, respectively. This implies that CH_3_CO* weakly adsorbs on the catalysts in the following order: Pd/Ce_2_Ti_2_O_7_–TiO_2_ < Pd < Pd/TiO_2_. As predicted by this model, the best-performing catalyst is Pd/Ce_2_Ti_2_O_7_–TiO_2_ followed by Pd and Pd/TiO_2_, supporting the experimental results.

From the electronic band structure in Figure 5a–c, it was evident that Pd/Ce_2_Ti_2_O_7_–TiO_2_ is electron-rich compared to Pd/TiO_2_ and Pd. Also, the separation (band gap) between the occupied energy states (valence band) and the unoccupied energy states (conduction band) was wide for Pd/TiO_2_ (poor conductor), while a zero gap was evident for both Pd (good conductor) and Pd/Ce_2_Ti_2_O_7_–TiO_2_ (highly conducting). This implies that less energy would be required to transport electrons from the valence band into the conduction band on the latter catalysts, particularly Pd/Ce_2_Ti_2_O_7_–TiO_2_. The projected density of states (PDOS) (Figure 5d–f) provides information on the electronic structure, showing atoms and specific orbitals (s, p, d, and f) that contribute to the different energy levels or bands within the catalyst materials. For Pd (Figure 5d), it is the d-orbital that dominates the energy levels; for Pd/TiO_2_ (Figure 5e) the p-orbital dominates the valence band, while the d-orbital the conduction band; and for Pd/Ce_2_Ti_2_O_7_–TiO_2_ (Figure 5f), it is the p-, d-, and f-orbitals that dominate both energy levels, with the f-orbital being more pronounced on the conduction band. This result confirms that the huge electronic band of the Pd/Ce_2_Ti_2_O_7_–TiO_2_ is due to multiorbital (p–d–f) interactions between the 3d, 2p, and 4f orbitals from Pd, TiO_2_, and Ce_2_Ti_2_O_7_, respectively, which endow the material with high conductivity.^90,108^ Interestingly, the electronic band structure and the PDOS excellently corroborate the experimental (EIS) data, in which the charge transport resistance decreased in the following order: Pd/TiO_2_ (27.12 kΩ) ≫ Pd/C > Pd/Ce_2_Ti_2_O_7_–TiO_2_ (0.553 kΩ), confirming the improved electron transfer properties on Pd/Ce_2_Ti_2_O_7_–TiO_2_ that accelerated EOR kinetics.

**Figure 5:**
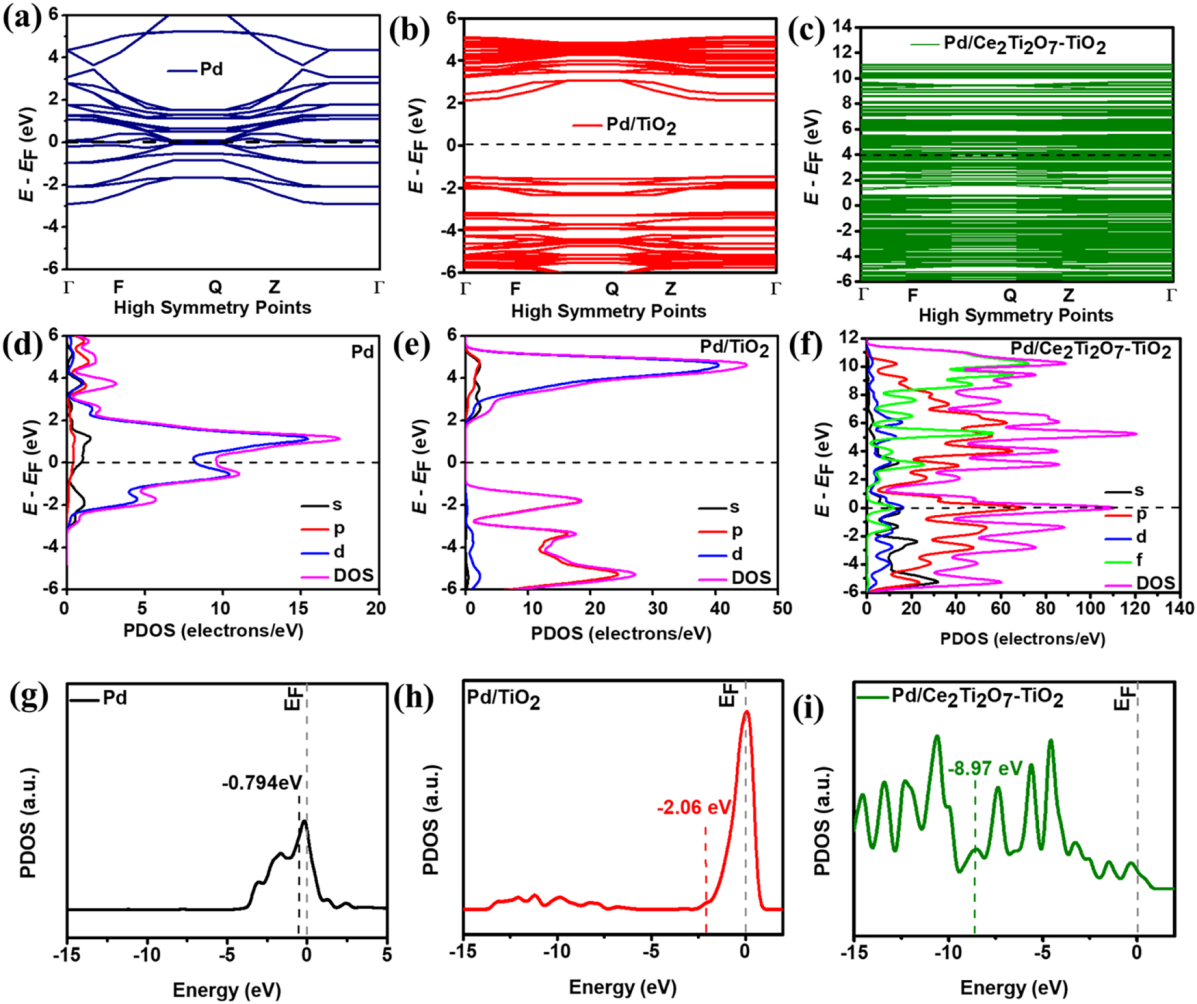
(a–c) Electronic band structure, (d–f) PDOS profiles, and (g–i) d-band structure of Pd, Pd/TiO_2_, and Pd/Ce_2_Ti_2_O_7_–TiO_2_.

The (ε_d_/eV) has emerged as an important descriptor for electrocatalytic activity. The ε_d_ of the supported Pd nanoparticles was calculated from the PDOS using eq 6:^105^

6 $$\varepsilon_{d}=\frac{\int \rho E\;dE}{\int \rho \;dE}$$

where ε_d_ was previously defined as the d-band center, ρ is the density of the d-band, *E* is the energy of the d-band, and ρ d*E* represents the number of states. In general, a downshifted ε_d_ (i.e., more negative ε_d_) is desirable in many electrocatalytic reactions as weak adsorption and subsequent easy desorption lead to faster reaction kinetics and improved catalyst stability.^55,85,109^ As shown in Figure 5g–i, the ε_d_ values for Pd, Pd/TiO_2_, and Pd/Ce_2_Ti_2_O_7_–TiO_2_ upon adsorption with CH_3_CO* were calculated as −0.794, −2.06, and −8.97 eV, respectively. Additionally, the ε_d_ of the pristine catalysts (Figure S13) followed the same trend, with Pd/Ce_2_Ti_2_O_7_–TiO_2_ (−8.89 eV) being more downshifted than Pd/TiO_2_ (−1.99 eV) and Pd (−0.480 eV). The downshifted ε_d_ of Pd/TiO_2_ and Pd/Ce_2_Ti_2_O_7_–TiO_2_ suggests optimal interactions between the reaction adsorbates and intermediates, leading to higher tolerance to catalyst poisoning, which was evident in the CO stripping and CA measurements.^96^

In summary, the enhanced electrocatalytic behavior of Pd/Ce_2_Ti_2_O_7_–TiO_2_ toward EOR relative to Pd/C was ascribed to, (i) the abundant O_V_ which plays a significant role toward the production of M–OH_ads_ that promotes the oxidation of reaction intermediate species, providing more active sites for the EOR and tolerance to CO-poisoning as demonstrated by the CO-strpping and CA test, (ii) the electronic modulation of Pd by Ce_2_Ti_2_O_7_–TiO_2_ as evidenced by XPS and DFT measurements resulted in a downshifted ε_d_ of Pd, favoring optimal adsorption and desorption of reactants, intermediate species, and products, endowing the catalyst with accelerated reaction kinetics and improved stability, (iii) the improved charge transfer properties as demonstrated by EIS and DFT measurements which showed improved electrode–electrolyte interfacial properties as well as an electron-rich electronic band structure due to the 3d–2p–4f orbital hybridization, and (iv) the improved stability attributed to the SMSI, which garnered the catalyst with reduced nanoparticle dissolution and/aggregation. On the other hand, despite Pd/TiO_2_ also containing O_V_ and M–OH_ads_, the catalysts’ inferior performance was attributed to the poor charge transport, as shown by the EIS data, which was further confirmed by the electronic band structure and PDOS data.

#### 3.2.3 Preliminary Results on the Amperometry-Based Sensor for Ethanol Using Pd/Ce_2_Ti_2_O_7_–TiO_2_

Owing to the satisfactory performance of Pd/Ce_2_Ti_2_O_7_–TiO_2_ catalyst for the electro-oxidation of ethanol, the application of Pd/Ce_2_Ti_2_O_7_–TiO_2_ as a nonenzymatic sensor was investigated, and the preliminary results in 0.5 M KOH are briefly discussed. The electrode preparation involved loading 0.025 mg of the catalyst (2.5 μg_Pd_) onto a screen-printed electrode suitable for point-of-care analysis. The CA measurements were first conducted in 0.5 M KOH (for blank readings), followed by spiked 0.5 M KOH in concentrations ranging from 2.0 to 94.0 mM. First, six measurements were conducted in the blank, followed by the addition of ethanol. As shown in Figure S14a, ethanol injection increased the response, and the corresponding calibration curve was constructed from triplicate readings at *t* = 5.0 s for each concentration and reported in Figure S14b. The calibration curve exhibited two linear concentration ranges, [2.0–10.0 mM] and [14.0–94.0 mM], with good linearity (*R* ^2^ = 0.991). The limit of detection (LoD) was determined using eq 7:

7 $$\mathrm{LoD}=\frac{3.3\times {\mathrm{SD}}_{\mathrm{blank}}}{m}$$

where SD_blank_ is the standard deviation of the blank, and *m* is the slope from the calibration curve. For the low and high concentration ranges, impressive LoD values of 12.0 and 77.1 μM were found, respectively. In comparison to similar works, like Pd/Ni/Si MCP electrode (16.8 μM),^110^ palladium paste nanocomposite electrode (20.0 μM)^66^ and Pd–Ni/SiNWs (10.0 μM),^66^ the LoD presented by our sensor was satisfactory. In South Africa, a blood alcohol concentration that is greater than 0.05 g per 100 mL of blood (10.85 mM) is considered illegal for driving.^67^ However, the application of the developed sensor to real samples such as blood and saliva was outside the scope of this work, but based on the low LoD presented herein, it would be worthwhile to investigate the electroanalysis in real samples such as blood and saliva in future studies. Not only does the Pd/Ce_2_Ti_2_O_7_–TiO_2_ catalyst show enhanced electrocatalytic behavior toward ethanol oxidation reactions in alkaline electrolyte, but it also holds promising applicability in a nonenzymatic amperometric sensor for ethanol.

## 4 Conclusions

In conclusion, TiO_2_ and Ce_2_Ti_2_O_7_–TiO_2_ supports were prepared, and a simple alcohol-reduction method was used to load Pd nanoparticles onto supports, resulting in Pd/TiO_2_ and Pd/Ce_2_Ti_2_O_7_–TiO_2_. Physical characterization, including XRD, SEM, HR-TEM, and EDX elemental mapping, confirmed the formation of Pd/TiO_2_ and Pd/Ce_2_Ti_2_O_7_–TiO_2_. The study showed the superior electrocatalytic performance of Pd/Ce_2_Ti_2_O_7_–TiO_2_ toward the ethanol oxidation reaction in an alkaline electrolyte, with a mass activity that was 2.70- and 2.63-fold higher than Pd/TiO_2_ and Pd/C, respectively. In comparison to Pd/C and Pd/TiO_2_, the enhanced electrocatalytic performance on Pd/Ce_2_Ti_2_O_7_–TiO_2_ was facilitated by the promotion of a bifunctional mechanism afforded by the Ce_2_Ti_2_O_7_–TiO_2_ O_V_-rich support, the metal-to-support interactions, which led to the modulation of the Pd d-band center in Pd/Ce_2_Ti_2_O_7_–TiO_2_, and the improved electron conductivity attributed to the multiorbital (3d–2p–4f) hybridization from the Pd, TiO_2_, and Ce_2_Ti_2_O_7_ in the Pd/Ce_2_Ti_2_O_7_–TiO_2_ catalyst. In addition to the superior electrocatalytic performance shown by Pd/Ce_2_Ti_2_O_7_–TiO_2_, the catalyst showed greater tolerance to poisoning than Pd/TiO_2_ and Pd/C, and an ECSA loss of only 18.8% compared to 33.3% for Pd/C, indicating improved stability. The preliminary results of Pd/Ce_2_Ti_2_O_7_–TiO_2_ as an amperometric nonenzymatic sensor for ethanol detection demonstrated a low LoD (12.0 μM) and high sensitivity (3.798 μA mM^–1^), illustrating its potential for low-concentration ethanol detection. This work sheds light on the synergistic effects of the heterostructure support, Ce_2_Ti_2_O_7_–TiO_2_, for Pd nanoparticles toward ethanol oxidation reaction in alkaline electrolyte. The catalyst Pd/Ce_2_Ti_2_O_7_–TiO_2_ shows promising results for its application as an anodic electrocatalyst for ethanol oxidation reactions in DEFCs and as an electrode platform in amperometric nonenzymatic sensors.

## Supplementary Material



## Notes

Additional experimental information, physicochemical characterization, and electrochemical measurements, all figures plotted with ORIGIN but pasted herein as pictures; these include the following: PXRD of TiO_2_-A; Raman analysis spectra for TiO_2_-A, TiO_2_, Ce_2_Ti_2_O_7_–TiO_2_; SEM images, and the corresponding EDX spectra for Pd/TiO_2_ and Pd/Ce_2_Ti_2_O_7_–TiO_2_; particle size distribution of Pd nanoparticles on Pd/TiO_2_ and Pd/Ce_2_Ti_2_O_7_–TiO_2_; HAADF-STEM and (b–d) EDX elemental mapping of Pd/TiO_2_; XPS survey spectra for TiO_2_-A, Pd/TiO_2_, and Pd/Ce_2_Ti_2_O_7_–TiO_2_; XPS Ti 2p and O 1s core levels for TiO_2_-A; CV profiles for Pd/TiO_2_, Pd/Ce_2_Ti_2_O_7_–TiO_2_, and Pd/C in 0.5 M KOH and CO-saturated 0.5 M KOH; LSV profiles collected at 10 mV s^–1^ in 0.5 M KOH + 0.5 M EtOH and the corresponding Tafel plots; CV curve of Pd/Ce_2_Ti_2_O_7_–TiO_2_ in 0.5 M KOH + 0.5 M EtOH collected at 50 mV s^–1^; potential sweeping at 100 mV s^–1^ in 0.5 M KOH for the catalysts; ECSA comparison after the 10th, 100th, 500th and 1000th CV cycles for Pd/Ce_2_Ti_2_O_7_–TiO_2_ and Pd/C in 0.5 M KOH; graphical presentation of the possible adsorption sites of the adsorbates, CH_3_CO*, and CHCO* on the surfaces of the investigated catalysts; PDOS profiles showing d-band centers of the bare Pd, Pd/TiO_2_, and Pd/Ce_2_Ti_2_O_7_–TiO_2_; chronoamperometric investigation at −0.3 V in 0.5 M KOH spiked with EtOH and the calibration curve for Pd/Ce_2_Ti_2_O_7_–TiO_2_; and table showing the fitted parameters of the Nyquist plots (PDF)
