# Supplementary material for: Synergistic Enhancement of Ethanol Oxidation on Pd/Ce2Ti2O7–TiO2 via Multiorbital (p–d–f) Interactions
Source: Inorg Chem. 2025 Dec 9;64(51):25129–42. doi: 10.1021/acs.inorgchem.5c04103 (PMC12754794; doi:10.1021/acs.inorgchem.5c04103)
Supplement: Supplementary file 1 [file ic5c04103_si_001.pdf]

Supporting Information

Synergistic Enhancement of Ethanol Oxidation on $Pd/Ce_2Ti_2O_7$-TiO_2 via Multi-Orbital (p–d–f) Interactions

Refiloe Modise [a,b], Patrick V. Mwonga [a], Jeseelan Pillay [b] and Kenneth I. Ozoemena[a]*

[a] Molecular Sciences Institute, School of Chemistry, University of the Witwatersrand, Private Bag 3, PO Wits, Johannesburg 2050, South Africa

[b] National Metrology Institute of South Africa, Private Bag X34, Lynnwood Ridge, 0040, South Africa

*Authors to whom correspondence should be addressed: K.I. Ozoemena (e-mail: Kenneth.ozoemena@wits.ac.za).

1. EXPERIMENTAL

1.1. Methods and Procedures: ***CAUTION!*** *We declare here that 'no uncommon hazards are noted' during the experimentation. However,* **caution** *is advised wherein every chemical reagent should be treated as poison which must never be ingested, and synthesis procedures carried out in fumehood.*

1.1.1. Physical Characterization

The average crystallite sizes of Pd nanoparticles were calculated using the Pd (1 1 1) miller index and the Scherrer's equation which is shown below.[1]

$$D = \frac{K\lambda}{\beta Cos\theta} \qquad \text{S1}$$

where D is the average crystallite size (nm) of Pd (1 1 1), K is shape factor constant (0.9 for spherical nanoparticles), λ is the wavelength of the X-ray radiation source (Cu = 0.154 nm), β is the full half width maximum (FHWM) of the diffraction peak in radians and θ is the Bragg angle.

1.1.2. Electrochemical Characterization

The electrochemical surface area (ECSA) of each electrocatalyst was calculated using eqn. S2.[2]

$$ECSA = \frac{Q}{m \times C} \qquad \text{S2}$$

where Q is the coulombic charge in mC measured from the palladium-oxide (Pd-O), m is the mass loading of Pd on each catalyst (2.5 μg_{Pd}) and C is the monolayer adsorbed oxygen on a poly Pd surface (0.405 $mCcm^{-2}$).

The constant phase impedance (Z_{CPE}) is defined as in eqn. S3.[3]

$$Z_{CPE} = \frac{1}{\sigma(j\omega)^{a}} \quad \text{S3}$$

where σ is a constant associated with the electrode-electrolyte interface, $j = \sqrt{-1}$, ω is the radial frequency, and a is the ideality factor with the following meanings: when a is -1, 0, 0.5 and 1, it implies that the Z_{CPE} behaves as an ideal inductor, resistor, Warburg impedance (Z_w) and ideal capacitor. Lastly, when $0.5 < a > 1$, then Z_{CPE} exhibits a pseudocapacitive behavior.

The Tafel slope equation is represented by

$$\eta = a + b\log j; \quad \text{S4}$$

and $$b = \frac{2.303\,RT}{\alpha nF} \quad \text{S5}$$

Where b is the Tafel slope ($mVdec^{-1}$), R is the ideal gas constant (8.314 J $mol^{-1}K^{-1}$), T is the temperature (298.15 K), α is the charge transfer coefficient and n is the number of electrons involved in the rate determining step (assumed to be two electrons in the formation of acetaldehyde from ethanol in this work) and F is the Faradaic constant (96485 C mol^{-1}).

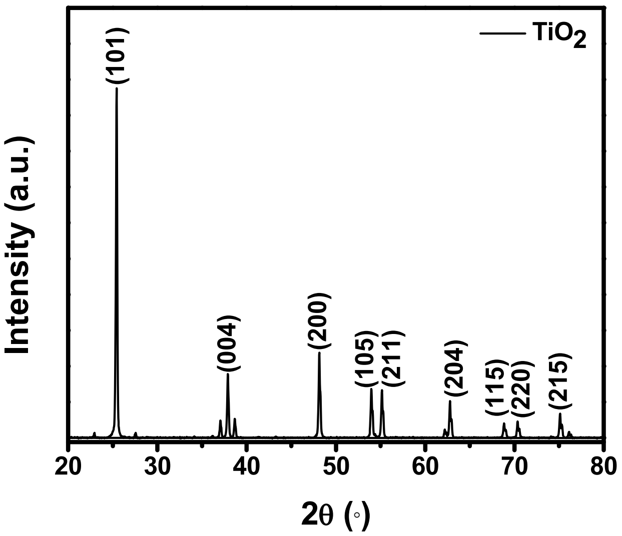

Figure S1. PXRD of TiO_2-A.

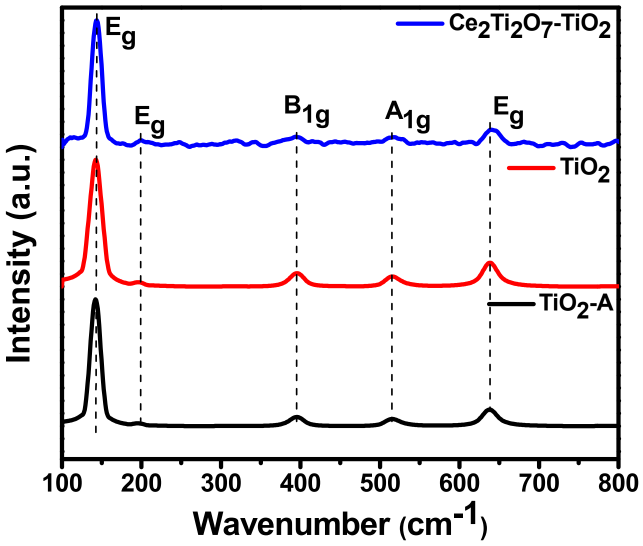

Figure S2. Raman analysis spectra for TiO_2-A, TiO_2, $Ce_2Ti_2O_7$-TiO_2.

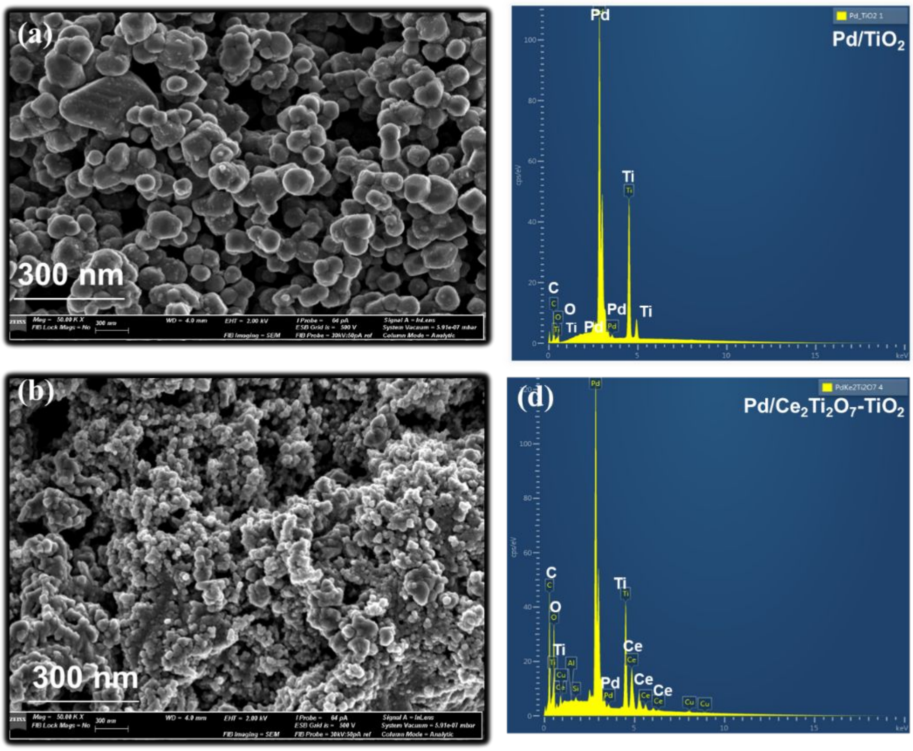

Figure S3. (a,b) SEM images and the corresponding (c,d) EDX spectra for Pd/TiO_2 and Pd/$Ce_2Ti_2O_7$-TiO_2, respectively.

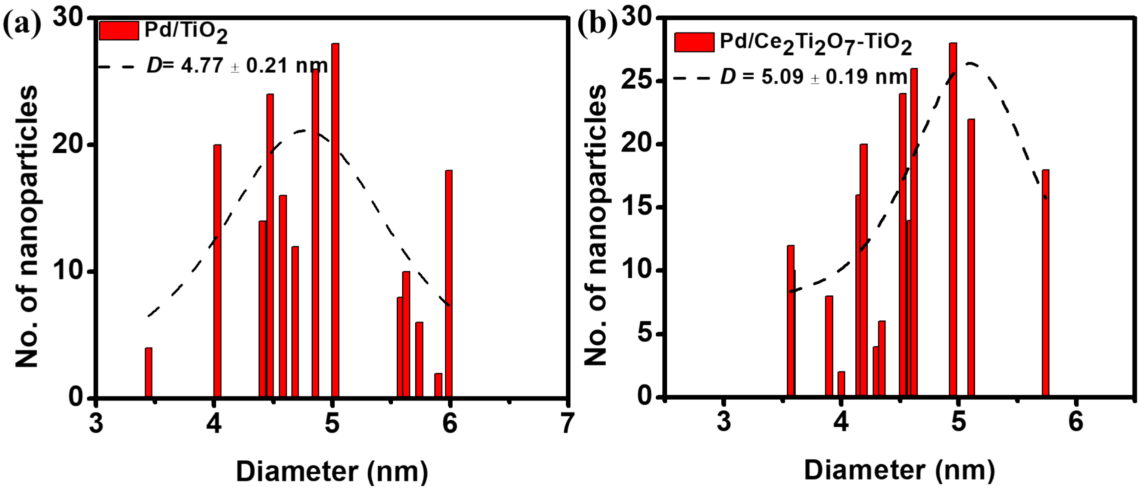

Figure S4. Particle size distribution of Pd nanoparticles on (a) Pd/TiO_2 and (b) $Pd/Ce_2Ti_2O_7$-TiO_2.

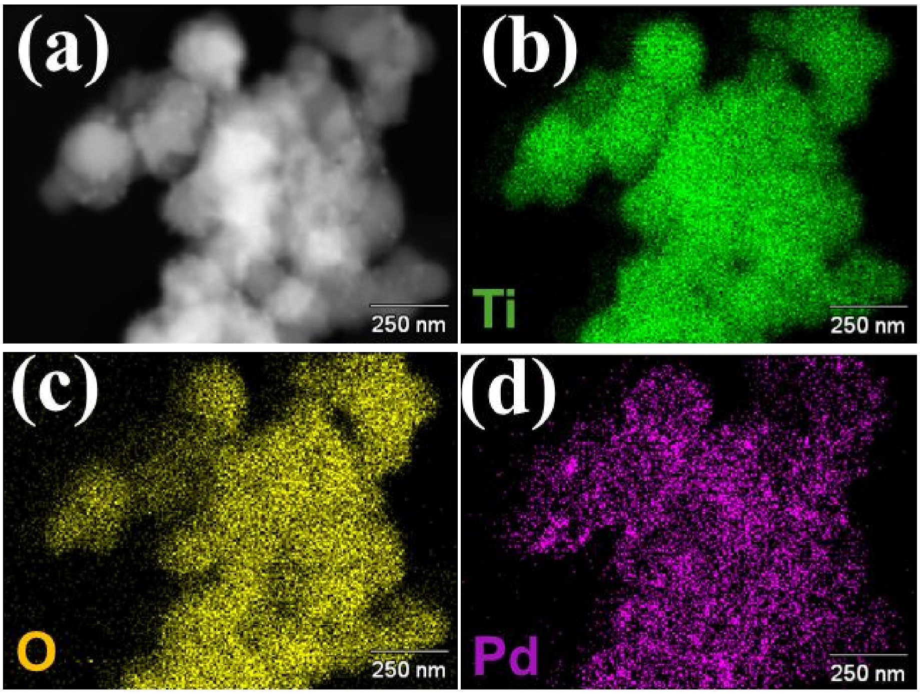

Figure S5. (a) HAADF-STEM and (b-d) EDX elemental mapping of Pd/TiO_2.

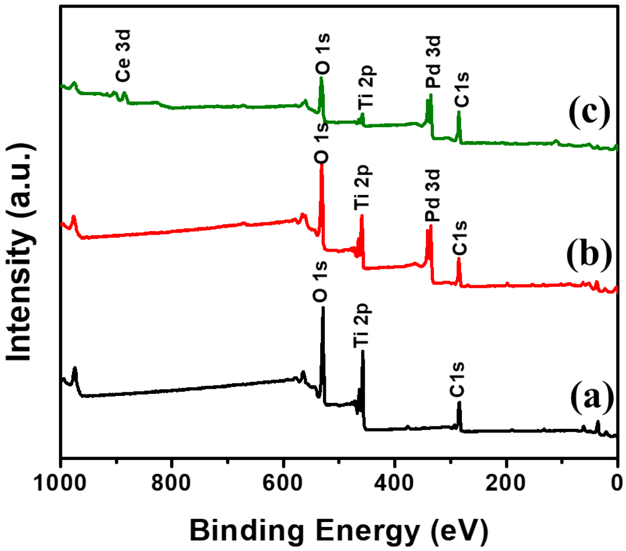

Figure S6. XPS survey spectra for (a) TiO_2-A, (b) Pd/TiO_2 and (c) Pd/$Ce_2Ti_2O_7$-TiO_2.

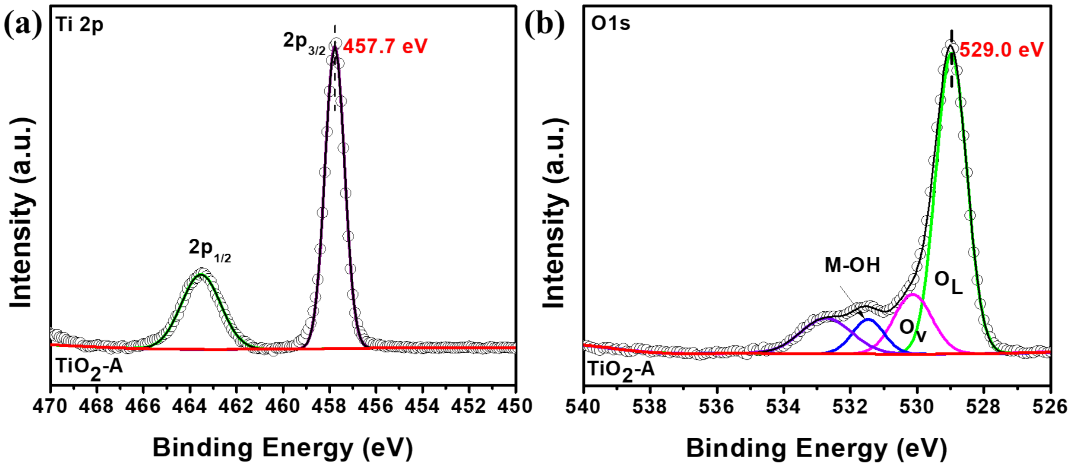

Figure S7. XPS (a) Ti 2p and (b) O1s core levels for TiO_2-A.

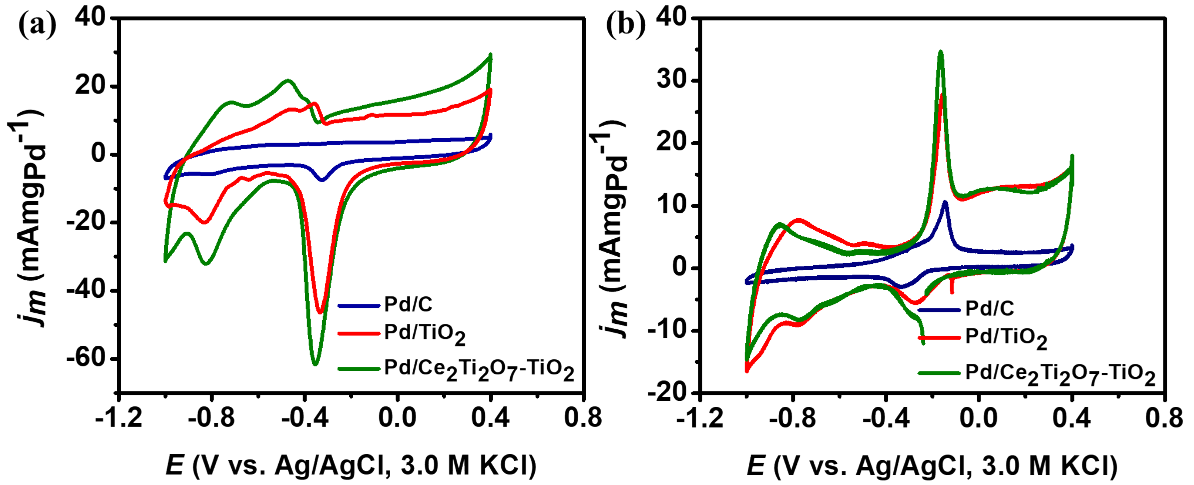

Figure S8. CV profiles for Pd/TiO_2, $Pd/Ce_2Ti_2O_7$-TiO_2 and Pd/C in (a) 0.5 M KOH and (b) CO-saturated 0.5 M KOH.

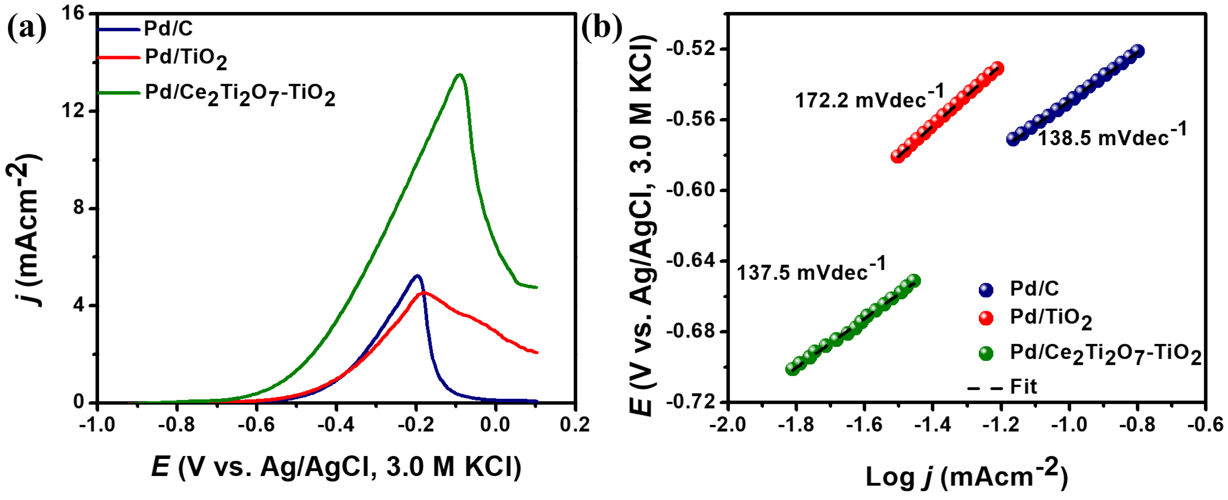

Figure S9. (a) LSV profiles collected at 10 mVs^{-1} in 0.5 M KOH + 0.5 M EtOH and (b) the corresponding Tafel plots.

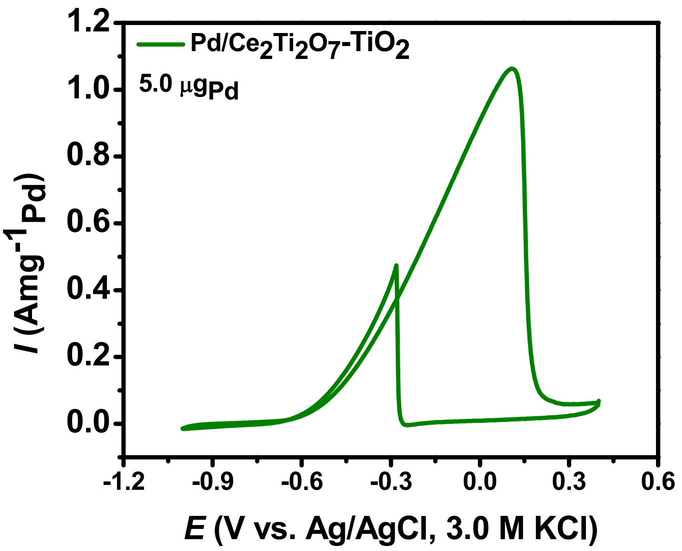

Figure S10. CV curve of Pd/$Ce_2Ti_2O_7$-TiO_2 in 0.5 M KOH + 0.5 M EtOH collected at 50 mVs^{-1}.

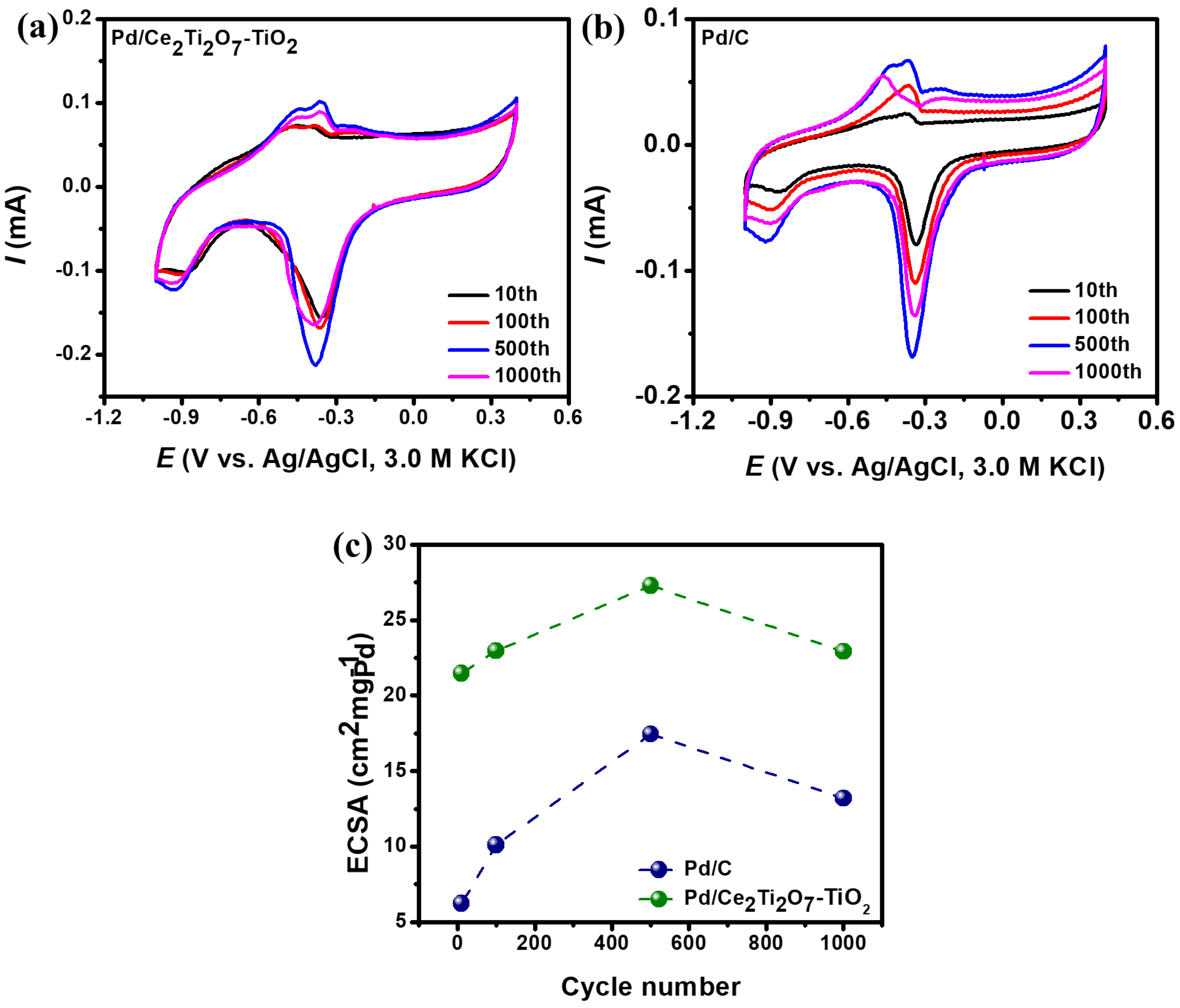

Figure S11. Potential sweeping at 100 mVs^{-1} in 0.5 M KOH for (a) Pd/$Ce_2Ti_2O_7$-TiO_2 and (b) Pd/C, *(c)* ECSA comparison after the 10th, 100th, 500th and 1000th CV cycles for Pd/$Ce_2Ti_2O_7$-TiO_2 and Pd/C in 0.5 M KOH.

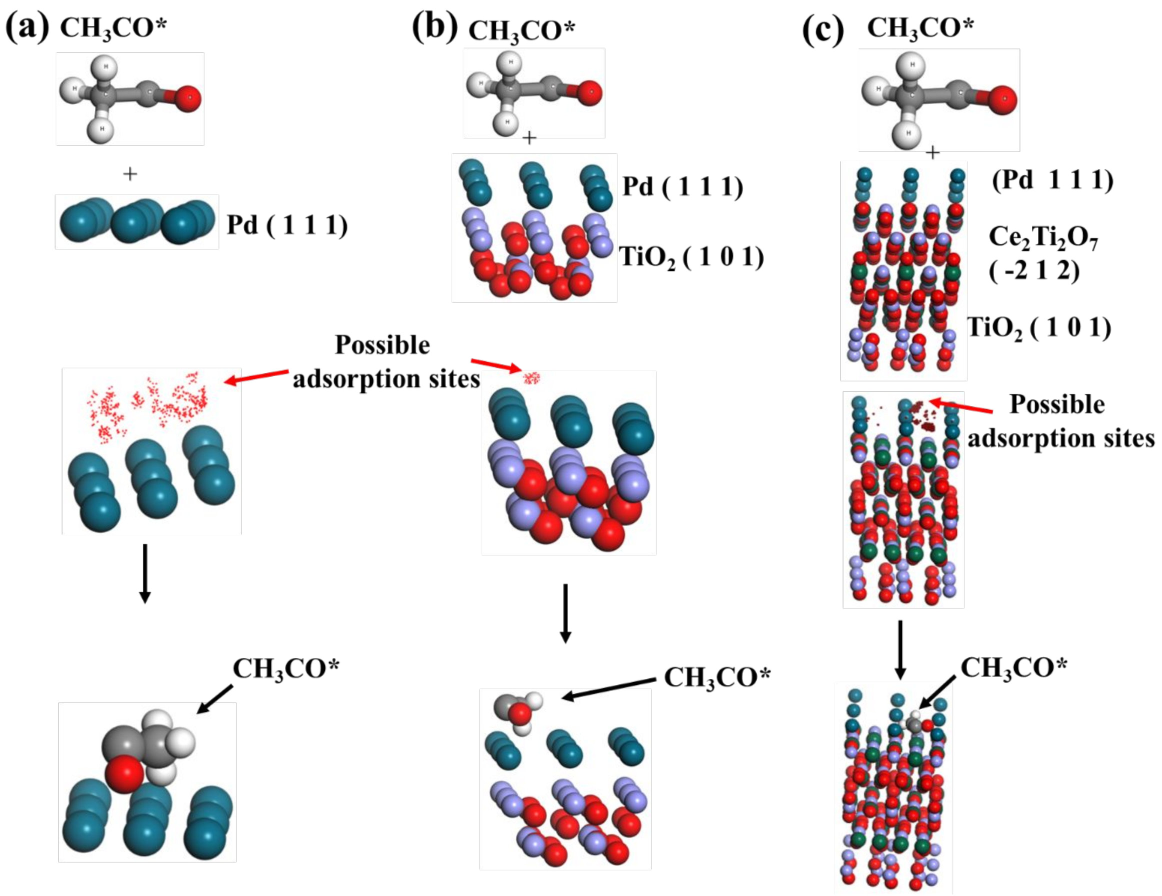

Figure S12. Graphical presentation of the possible adsorption sites of the adsorbates, CH_3CO*, and CHCO* on (a) Pd, (b) Pd/TiO_2, and (c)Pd/$Ce_2Ti_2O_7$-TiO_2.

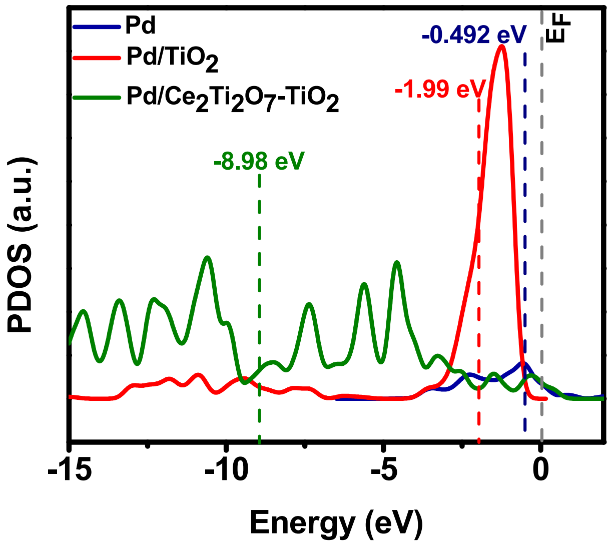

Figure S13. PDOS profiles showing d-band centers of the bare Pd, Pd/TiO_2 and Pd/$Ce_2Ti_2O_7$-TiO_2.

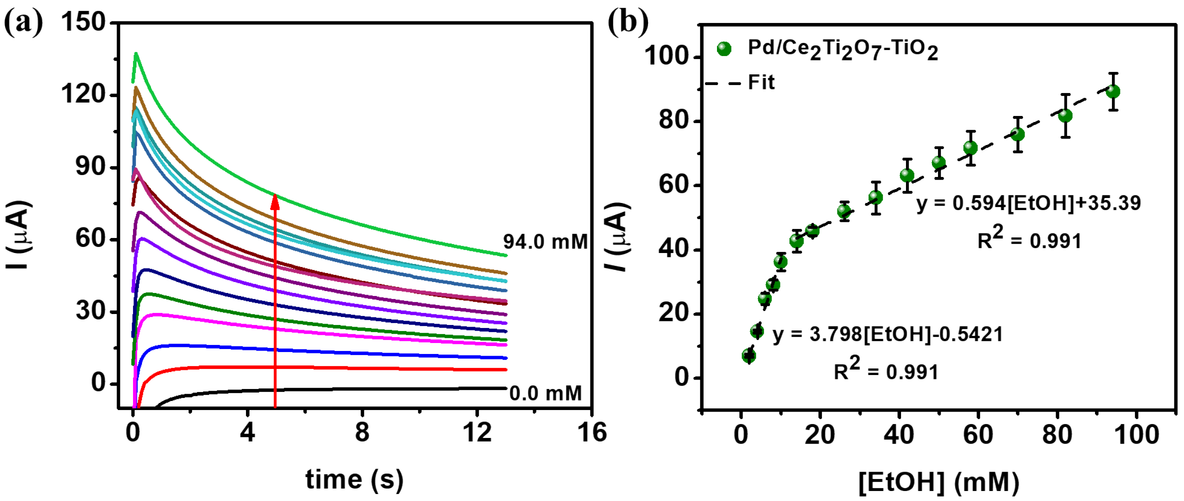

Figure S14. (a) CA at -0.3V in 0.5 M KOH spiked with EtOH [0 – 94.0 mM] and (b) the calibration curve for Pd/$Ce_2Ti_2O_7$-TiO_2.

Table S1. The EEC values obtained from the fitted Nyquist plots.

**Electrocatalyst**	R_s (Ω)	*CPE* (μFs^{a-1})	R_{int} (kΩ)	*CPE* (μFs^{a-1})	R_{ct} (kΩ)	R_{tot} (kΩ)
**Pd/TiO_2**	0.132 ± 0.0138	26.2 ± 6.12	1.09 ± 0.146	56.5 ± 2.54	26.0 ± 1.06	27.1
**Pd/$Ce_2Ti_2O_7$-TiO_2**	0.0074 ± 0.483 × 10^{-3}	120.6 ± 8.51	0.150 ± 0.926 × 10^{-3}	0.0185 ± 1.22	0.404 ± 0.0102	0.553
**Pd/C**	0.00450 ± 0.177 × 10^{-3}	0.0495 ± 0.00458	0.163 ± 0.711 × 10^{-3}	57.84 ± 1.25	4.63 ± 0.0865	4.79

REFERENCES

1. M. K. Manglam, S. Kumari, J. Mallick and M. Kar, Crystal structure and magnetic properties study on barium hexaferrite of different average crystallite size, *Appl. Phys. A*, 2021, **127**, 138.
2. K. Z. Mousaabadi, A. A. Ensafi, T. R. Adriyani and B. Rezaei, Pd/Hemin-rGO as a bifunctional electrocatalyst for enhanced ethanol oxidation reaction in alkaline media and hydrogen evolution reaction in acidic media, *Int. J. Hydrogen Energy*, 2023, **48**, 21259–21269.
3. T. J. Ehirim, O. C. Ozoemena, P. V. Mwonga, A. B. Haruna, T. P. Mofokeng, K. De Wael and K. I. Ozoemena, Onion-like Carbons Provide a Favorable Electrocatalytic Platform for the Sensitive Detection of Tramadol Drug, *ACS Omega*, 2022, **7**, 47892–47905.
